# Targeting RAGE prevents muscle wasting and prolongs survival in cancer cachexia

**DOI:** 10.1002/jcsm.12561

**Published:** 2020-03-11

**Authors:** Sara Chiappalupi, Guglielmo Sorci, Aleksandra Vukasinovic, Laura Salvadori, Roberta Sagheddu, Dario Coletti, Giorgia Renga, Luigina Romani, Rosario Donato, Francesca Riuzzi

**Affiliations:** ^1^ Department of Experimental Medicine University of Perugia Perugia Italy; ^2^ Department of Anatomical, Histological, Forensic and Orthopedic Sciences Sapienza University of Rome Rome Italy; ^3^ CNRS UMR 8256, INSERM ERL U1164, Biological Adaptation and Aging B2A Sorbonne Université Paris France; ^4^ Interuniversity Institute of Myology Perugia Italy; ^5^ Centro Universitario di Ricerca sulla Genomica Funzionale University of Perugia Perugia Italy

**Keywords:** Cancer cachexia, RAGE, Inflammation, Muscle atrophy, S100B, HMGB1, Cytokines, Myogenin

## Abstract

**Background:**

Cachexia, a multifactorial syndrome affecting more than 50% of patients with advanced cancer and responsible for ~20% of cancer‐associated deaths, is still a poorly understood process without a standard cure available. Skeletal muscle atrophy caused by systemic inflammation is a major clinical feature of cachexia, leading to weight loss, dampening patients' quality of life, and reducing patients' response to anticancer therapy. RAGE (receptor for advanced glycation end‐products) is a multiligand receptor of the immunoglobulin superfamily and a mediator of muscle regeneration, inflammation, and cancer.

**Methods:**

By using murine models consisting in the injection of colon 26 murine adenocarcinoma (C26‐ADK) or Lewis lung carcinoma (LLC) cells in BALB/c and C57BL/6 or *Ager* ^−/−^ (RAGE‐null) mice, respectively, we investigated the involvement of RAGE signalling in the main features of cancer cachexia, including the inflammatory state. *In vitro* experiments were performed using myotubes derived from C2C12 myoblasts or primary myoblasts isolated from C57BL/6 wild type and *Ager* ^−/−^ mice treated with the RAGE ligand, S100B (S100 calcium‐binding protein B), TNF (tumor necrosis factor)α±IFN (interferon) γ, and tumour cell‐ or masses‐conditioned media to analyse hallmarks of muscle atrophy. Finally, muscles of wild type and *Ager* ^−/−^ mice were injected with TNFα/IFNγ or S100B in a tumour‐free environment.

**Results:**

We demonstrate that RAGE is determinant to activate signalling pathways leading to muscle protein degradation in the presence of proinflammatory cytokines and/or tumour‐derived cachexia‐inducing factors. We identify the RAGE ligand, S100B, as a novel factor able to induce muscle atrophy *per se* via a p38 MAPK (p38 mitogen‐activated protein kinase)/myogenin axis and STAT3 (signal transducer and activator of transcription 3)‐dependent MyoD (myoblast determination protein 1) degradation. Lastly, we found that in cancer conditions, an increase in serum levels of tumour‐derived S100B and HMGB1 (high mobility group box 1) occurs leading to chronic activation/overexpression of RAGE, which induces hallmarks of cancer cachexia (i.e. muscle wasting, systemic inflammation, and release of tumour‐derived pro‐cachectic factors). Absence of RAGE in mice translates into reduced serum levels of cachexia‐inducing factors, delayed loss of muscle mass and strength, reduced tumour progression, and increased survival.

**Conclusions:**

RAGE is a molecular determinant in inducing the hallmarks of cancer cachexia, and molecular targeting of RAGE might represent a therapeutic strategy to prevent or counteract the cachectic syndrome.

## 1 Introduction

Cachexia is a highly debilitating multifactorial syndrome affecting more than 50% of patients with advanced cancer, especially lung and upper‐gastrointestinal cancer. Although cachexia directly accounts for about 20% of all cancer‐associated deaths, it remains an underestimated and poorly understood process, for which a complete pharmacological cure is not available. The major clinical feature of cachexia is skeletal muscle atrophy that leads to pronounced weight loss, drastically dampened patients' quality of life, reduced response and tolerance to chemotherapy, and poor prognosis and outcome.^1^, ^2^

Systemic inflammation is thought to be a major mediator of muscle wasting in individuals affected by cancer. Several key cachexia‐inducing factors have been shown to be produced by host immune cells in response to the tumour [e.g. interferon (IFN)γ, tumour necrosis factor (TNF)α, and interleukin (IL)‐6] or by the tumour cells themselves (e.g. myostatin, activins, GDF15, TWEAK).^2^, ^3^, ^4^ These factors induce a catabolic state in skeletal muscle principally caused by the activation of the ubiquitin–proteasome system (UPS) and autophagy–lysosome system (ALS), as well as a decrease of protein synthesis.^1^, ^3^ In cancer conditions, several pathways [i.e. phosphatidylinositol (PI)‐3‐kinase/protein kinase B (Akt)/mammalian target of rapamycin (mTOR), p38 mitogen‐activated protein kinase (MAPK), extracellular‐signal‐regulated kinases (ERK 1/2), and c‐Jun N‐terminal kinase (JNK)] and transcription factors [such as signal transducer and activator of transcription 3 (STAT3) and nuclear factor kappa B (NF‐κB)] are involved in the induction of the muscle‐specific ubiquitin ligases, atrogin‐1 (muscle atrophy F‐box protein; *Fbxo32*) and muscle RING finger‐1 (*Trim63*), known as atrogenes, leading to proteolysis, especially of sarcomeric myosin heavy chain (MyHC).^5^, ^6^ Interestingly, reports revealed an unexpected link between myogenin, an essential transcription factor in the regulation of muscle differentiation,^7^ and the maximal induction of atrogenes in several non cancer conditions leading to muscle wasting.^8^, ^9^

RAGE (receptor for advanced glycation end‐products) is a multiligand receptor of the immunoglobulin superfamily considered as a key mediator of several physiological and pathological processes through the activation of multiple cellular signalling cascades, depending on the cell type, environmental cues (i.e. the presence and concentration of RAGE ligands), and the density/activity of the receptor on the cell surface.^10^, ^11^, ^12^ RAGE and its ligands, high mobility group box 1 (HMGB1) and the S100 calcium‐binding protein B (S100B), have been implicated in cell proliferation and survival, autophagy, invasiveness, metastasis, and angiogenesis in several cancer types.^13^, ^14^, ^15^ S100B is normally present in human serum, and its serum levels are elevated in some human tumours, representing a prognostic marker in patients with cutaneous melanoma and breast cancer.^6^ Moreover, by interacting with the ‘alarmins’ HMGB1 and S100B, RAGE participates in innate and adaptive immune responses, immune cell migration and chemotaxis, and cytokines production.^11^

We have identified RAGE, S100B, and HMGB1 as physiological regulators of myogenesis and muscle regeneration upon acute muscle injury via p38 MAPK‐dependent induction of myogenin.^12^, ^16^, ^17^, ^18^ However, the continuous release of high S100B and HMGB1 by damaged myofibres and infiltrating macrophages as found in muscular dystrophy fuels inflammation and dampens the reparative process,^17^, ^19^ and overstimulation of RAGE leads to muscle wasting and altered muscle metabolism in ageing and pathological conditions such as diabetes, obesity, and myopathies.^12^, ^20^ Yet, the possible role of RAGE and its ligands, in particular S100B, in cancer‐induced muscle wasting has not been investigated so far. Herein, we show that (i) inflammatory cytokines and cachexia‐inducing factors (including S100B and HMGB1) induce RAGE expression in skeletal muscles; and (ii) RAGE hyperactivation plays a relevant role in the loss of muscle mass in cancer conditions principally via a p38 MAPK/myogenin/atrogin‐1 axis. We propose that RAGE is an important molecular determinant of muscle atrophy in cancer patients and a potential therapeutic target.

## 2 Methods

### 2.1 Cell cultures

Murine C2C12 myoblasts were grown in high‐glucose Dulbecco's Modified Eagle's Medium (DMEM) supplemented with 20% foetal bovine serum (FBS), 100 U/ml penicillin and 100 mg/ml streptomycin (P/S) (growth medium, GM). Differentiation into myotubes was induced by shifting sub‐confluent myoblasts to DMEM supplemented with 2% horse serum (HS) (differentiation medium, DM) for 4 days. Primary myocytes were obtained as described.^16^

Myotubes were treated with TNFα (20 ng/ml) ± IFNγ (100 U/ml), S100B (0–20 μg/ml), or HMGB1 (0–30 μg/ml) for the indicated time. In parallel experiments, myotubes were pre‐treated for 30 min with anti‐RAGE blocking antibody (0**‐50** μg/ml), a neutralizing anti‐S100B antibody (2 μg/ml), glycyrrhizin (50 μM) or the chemical inhibitors, SB 203580 (inhibitor of p38 MAPK; 10 μM) or BAY 11‐7082 (inhibitor of NF‐κB; 10 μM).

Lewis lung carcinoma (LLC) and colon 26 murine adenocarcinoma (C26‐ADK) cells were cultured in high‐glucose DMEM or Roswell Park Memorial Institute (RPMI) 1640, respectively, containing P/S and supplemented with 10% FBS (GM). C2C12, LLC, and C26 cells were maintained in a humidified atmosphere containing 5% CO_2_ at 37 °C. To obtain conditioned media (CM), once the plates reached a > 90% confluence, GM was removed, and cells were washed twice with sterile phosphate‐buffered saline (PBS) and incubated in serum‐free DMEM with P/S. After 24 h, the medium was collected and centrifuged in 50 ml tubes at 4500 r.p.m for 15 min at 4°C. Aliquots of the cell‐cleared medium were stored at −80°C. To obtain tumour‐conditioned medium (TM), LLC and C26 masses were dissected and placed in sterile PBS. The tumour was minced into parts of approximately 2 mm^3^, washed twice with PBS and incubated in serum‐free DMEM containing P/S. TM was collected after a 48 h incubation. C2C12 myotubes were treated with CM or TM added to a differentiation medium (25% v/v) for the indicated time.

### 2.2 Animal models

Ten‐week‐old mice were injected subcutaneously (s.c.) in the hind flank with LLC cells (1.0 × 10^6^ cells/mouse) in the case of C57BL/6 wild type (WT) and *Ager* ^−/−^ mice or with C26 cells (0.5 × 10^6^ cells/mouse) in the case of BALB/c mice. Tumours were allowed to develop for different time‐points, compared with PBS‐injected control mice. Body weight was measured every 3 days starting from tumour appearance and calculated by subtracting tumour weight from total weight. Tumour measurements were taken using a digital calliper (Exacta Optech) and tumour volumes calculated with the formula, (length × width^2^)/2. At the indicated time, animals were sacrificed and sera were collected. Skeletal muscles, adipose tissue, lungs, spleens, and tumours were surgically excised and weighed. In any case, mice were sacrificed if human‐end points were reached. The occurrence and numbers of lung metastases were measured.

*Gastrocnemius* (GC) muscles of WT and *Ager* ^−/−^ mice were injected with PBS, or TNF‐α (3 μg/muscle) plus IFNγ (5000 U/muscle) or S100B (50 ng/muscle) for three consecutive days. To obtain CM, muscles were incubated in PBS for 1 h at 4°C.

Treatments of animals were performed under zolazepam/tiletamine or isofluorane anaesthesia. Animal procedures followed the 3Rs principles in alignment with the Directive 2010/63/EU of the European Union and approved by the Ethics Committee of Perugia University and Italian Ministry of Health. Mice were housed under specific pathogen‐free conditions on a 12 h light/day cycle, and raised under a standard mouse diet.

### 2.3 Kondziela's inverted screen test

Each mouse was placed in the centre of a wire mesh screen, the screen was rotated by 180°, and the time when the mouse fell off was measured for a maximum of 8 min. Each mouse was evaluated in three trials at 10 min inter‐trial intervals.

### 2.4 Real‐time polymerase chain reaction (real‐time PCR)

Total RNA was extracted from cell cultures or skeletal muscle homogenates using a commercial TRIsure™ reagent following the manufacturer's instructions. Reverse‐transcription was obtained using a PrimeScript™ RT reagent kit. Real‐time PCR analyses of messenger RNA (mRNA) contents were performed on Stratagene Mx3000P (Agilent Technologies, CA, USA) using 5×HOT FIREPol EvaGreen qPCR Mix Plus (ROX) ready‐to‐use solution. Calculation was performed with a specific software in comparison with a standard gene (*Gapdh*). The primers used for real‐time PCR analysis are reported in *Table* *S2* (Supplemental Material).

### 2.5 Western blot (WB)

Myotube cultures, skeletal muscle or tumour masses homogenates were lysed in protein extraction buffer containing 10 mM Tris–HCl (pH 7.4), 2.5% v/v sodium dodecyl sulfate, 100 mM dithiothreitol, and 200 mM phenylmethanesulfonyl fluorid, 10 mg/ml aprotinin, 1 mg/ml pepstatin, and 5 mg/ml leupeptin, all from Sigma Aldrich. Equal amounts of total protein extract were resolved by sodium dodecyl sulfate–polyacrylamide gel electrophoresis and transferred to nitrocellulose blots (Amersham™ Protran™, 0.45 μm). Following blocking with 5% non‐fat dried milk primary and secondary antibodies were applied as indicated in *Table* *S3* (Supplemental Material). For S100B detection, blots were blocked with 5% bovine serum albumin (BSA). The immune reactions were developed by enhanced chemiluminescence. C‐DiGit Blot Scanner (LI‐COR, NE, USA) was used for blot analysis.

### 2.6 Immunocytochemistry (ICC) and immunofluorescence (IF)

Myotubes were washed with Tris‐buffered saline (TBS), fixed for 7 min with cold absolute methanol and permeabilized for 10 min with 0.1% Triton X‐100 in TBS solution, blocked with blocking buffer (10% HS and 0.1% Triton X‐100 in TBS) for 1 h, and incubated overnight at 4°C with mouse anti‐MyHC‐II primary antibody (1:1000 in 10% HS). Myotubes were washed, incubated with appropriate secondary biotinilated‐antibody, rinsed with TBS containing 0.01% Tween‐20 (T‐TBS), and then incubated for 45 min with Vectastain ABC reagents. After washing with TBS, myotubes were incubated with 0.01% 3, 3‐diaminobenzidine tetrahydrochloride, Nichel, and 0.006% H_2_O_2_ in 50 mM Tris–HCl (pH 7.4) for 8 min. The reaction was blocked with 50 mM Tris–HCl. Mounting medium (Dako Corporation) was added, and pictures were taken using a bright field microscope (Olympus BX51) equipped with a digital camera.

For IF staining, myotubes cultivated on sterile glass coverslips were fixed with 4% paraformaldehyde, permeabilized using 0.1% Triton X‐100 in PBS, blocked with blocking buffer containing 1% glycine and 3% BSA in PBS, and incubated in a humid chamber overnight at 4 °C with mouse anti‐MyHC‐II or rabbit anti‐pho‐STAT3 primary antibody in PBS containing 3% BSA (*Table* *S4* in Supplemental Material). The next day, coverslips were incubated with appropriate Alexa Fluor 488‐conjugated antibody in PBS containing 3% BSA (*Table* *S4* in Supplemental Material), in a light‐tight humid chamber, and counterstained with 4′,6‐diamidino‐2‐phenylindole (DAPI) to visualize the nuclei. At the end, coverslips were mounted with fluorescent mounting medium containing 80% glycerol and 20% PBS and viewed by an epifluorescence microscope (Leica DMRB) equipped with a digital camera.

### 2.7 Evaluation of myotube diameter

Myotube diameters were determined on images of MyHC‐II staining at 20× magnification using ImageJ software. Three different measurements were performed along the longitudinal axis of each myotube. Average diameters of at least 100 myotubes from 10 randomly chosen fields for each condition were determined. Results were expressed as percentages with respect to control myotubes**.**

### 2.8 Analysis of conditioned media

CM or TM were added with 1/100 volume of 2% sodium deoxycholate (Sigma Aldrich) and subjected to precipitation with 1/10 volume of 100% trichloroacetic acid (Sigma Aldrich). The resultant pellets were resuspended in Laemmli buffer and titrated with 1 N NaOH to obtain the normal blue colour of the sample buffer, boiled for 5 min, and subjected to western blotting to analyse the expression of S100B or HMGB1 (*Table* *S3* in Supplemental Material).

### 2.9 Histology, immunohistochemistry (IHC), and IF

Muscles, spleens, lungs, and tumour masses were isolated, formalin‐fixed, and paraffin‐embedded in order to maximally preserve morphology. Muscle cross‐sections measuring 4 μm were obtained and processed for standard haematoxylin/eosin staining. Tumour masses were processed for Masson's trichrome staining (Sigma‐Aldrich) to evaluate fibrosis. Slices were analysed and photographed with a bright field microscope (Olympus BX51) equipped with a digital camera. Myofiber cross‐sectional areas (CSAs) and tumour necrosis areas were measured using ImageJ software on total sections at 100 μm intervals for each muscle or tumour mass.

Paraffin sections were deparaffinized with xylene and rehydrated in a graded ethanol series. Antigen retrieval was performed by boiling for 1.5 h in 10 mM citric acid buffer (pH 6.0), and depletion of endogenous peroxidase was accomplished by treatment with 3% H_2_O_2_. The sections were probed with anti‐RAGE primary antibody diluted 1:50 in B.B. (T‐TBS and 10% HS) and the sections incubated overnight in a humid chamber at 4°C. After several washings in T‐TBS, the sections were incubated with anti‐goat biotinylated antibody (1:500 dilution) for 1 h in B.B. The sections were rinsed in T‐TBS, incubated for 45 min with Vectastain ABC reagents, washed again in TBS, and incubated with 0.01% 3‐diaminobenzidine tetrahydrochloride (DAB), 0.006% H_2_O_2_ in 50 mM Tris–HCl (pH 7.4). Nuclei were counterstained with haematoxylin. The sections were dehydrated and mounted with EuKitt mounting medium (Electron Microscopy Sciences). Slices were photographed in a bright field microscope (Olympus BX51) equipped with a digital camera.

To perform double IF , after cooling, slices were washed in PBS incubated for 30 min in 1% H_2_O_2_ in methanol in the dark to inactivate endogenous peroxidase, blocked for 1 h at room temperature with B.B. (1% BSA, 0.4% Triton X‐100, 10% HS), and incubated in a humid chamber overnight at 4°C with the primary antibodies (*Table* S4 in Supplemental Material) diluted in 1% BSA and 0.4% Triton X‐100 in PBS. The next day, slices were washed in Triton X‐100 in PBS and incubated with the specific Alexa Fluor‐conjugated antibodies (*Table* S4 in Supplemental Material) diluted 1:100 in 0.1% BSA in PBS. To reduce autofluorescence, the sections were additionally stained with 0.3% Sudan Black B (Sigma Aldrich) in 70% ethanol for 10 min in the dark. For RAGE and MAC3, double immunofluorescence slices were blocked with 3% BSA and 1% glycine in PBS, and primary and secondary antibodies diluted in 3% BSA in PBS. Nuclei were counterstained with DAPI. After rinsing, sections were mounted with anti‐fading mounting medium (Dako Corporation, Denmark) and viewed in an epifluorescence microscope (Leica DMRB) equipped with a digital camera.

### 2.10 Enzyme‐linked immunosorbent assay (ELISA) and multiplex cytokine assay

Blood samples were collected after mice terminal decapitation, and serum was collected. Serum cytokine levels were measured using a ProTM Mouse Cytokine 23‐plex Assay kit according to the manufacturer's instructions and analysed by a MAGPIX® Multiplexing Instrument (Luminex Corporation). Serum S100B and HMGB1 levels were measured using a mouse‐specific ELISA assay kits according to the manufacturers' protocol.

### 2.11 Reagents and resources

See Supporting Information Table *S1*.

### 2.12 Statistical analysis

Quantitative data are presented as means ± standard error (SEM) of the mean or standard deviation (SD) of at least three independent experiments. The number of animals used is specified in each experiment. Counts were performed by three independent operators blind to the treatments. Representative experiments and images are shown unless stated otherwise. Statistical analysis was performed using two‐tailed, unpaired *t*‐test for single variables and two‐way analysis of variance followed by Dunnet test for multiple variables. A Mann–Whitney test was used for statistical analysis of lung metastasis numbers. *P* values lower than 0.05 were considered statistically significant. All statistical data were processed by IBM® SPSS® Statistics Version 18 software.

## 3 Results

### 3.1 Tumour presence causes re‐expression of RAGE in muscle tissue and increases serum levels of the RAGE ligands, S100B, and HMGB1

RAGE is absent in healthy adult myofibers and quiescent muscle stem cells (i.e. satellite cells; SCs). However, RAGE is expressed during skeletal muscle development, and it is transiently re‐expressed in activated/proliferating SCs, differentiating myoblasts and regenerating myofibers upon muscle injury.^16^, ^21^ Re‐expression of RAGE in adult myofibers occurs also in certain myopathies.^12^

**Figure 1:**
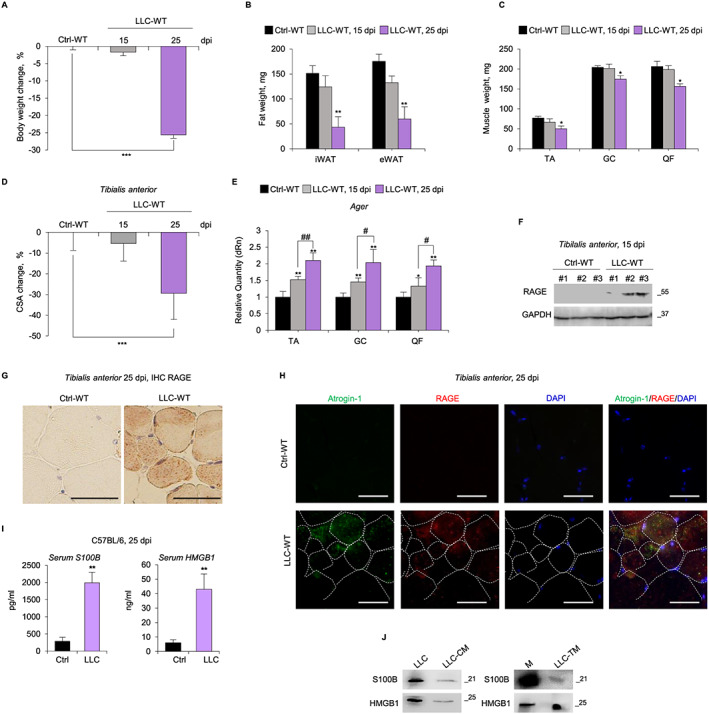
Tumour presence causes a re‐expression of receptor for advanced glycation end‐products (RAGE) in muscle tissue and increases serum levels of the RAGE ligands, S100 calcium‐binding protein B (S100B) and high mobility group box 1 (HMGB1). (*A–I*) C57BL/6 wilde‐type (WT) mice injected with Lewis lung carcinoma (LLC‐WT) cells or vehicle (Ctrl‐WT) (*n* = 8 each group) were sacrificed at the indicated day post‐injection (dpi). The weights of body (*A*), fat (i.e. inguinal white adipose tissue, iWAT and epididymal white adipose tissue, eWAT) (*B*), and *tibialis anterior* (TA)*, gastrocnemius* (GC) and *quadriceps femoris* (QF) muscles (*C*) were measured. (*D*) Reported is the average of percentage changes of TA myofiber cross‐sectional area (CSA) for LLC‐bearing mice compared with control mice (see also *Figure* S1A). Skeletal muscles were analysed for RAGE expression by real‐time PCR (*E*), western blot (WB) (*F*), and immunohistochemistry (IHC) (*G*). (*H*) RAGE (*red*) was detected by immunoflourescence (IF) in atrophic myofibers marked with atrogin‐1 (*green*). 4′,6‐diamidino‐2‐phenylindole (DAPI) (*blue*) was used to stain nuclei. Dashed lines indicated myofiber borders. (*I*) Serum levels of S100B and HMGB1 were measured by enzyme‐linked immunosorbent assay (ELISA). (*J*) S100B and HMGB1 were detected in LLC cell lysates, conditioned medium derived from LLC cells (LLC‐CM), and LLC tumour masses (LLC‐TM) by WB. M, purified S100B (5 ng), or HMGB1 (10 ng). Results are means ± standard error of the mean. Statistical analysis was conducted using the two‐tailed *t*‐test. ^*^ *P* < 0.05, ^**^ *P* < 0.01, and ^***^ *P* < 0.001 significantly different from control mice. ^#^ *P*<0.05, ^##^ *P*<0.01 significantly different. Scale bars (*G*,*H*), 50 μm.

We analysed RAGE expression over time in *tibialis anterior* (TA), GC, and *quadriceps femoris* (QF) muscles of C57BL/6 mice injected s.c. with LLC cancer cells (LLC‐WT), as one of the best‐characterized experimental models of cancer cachexia.^22^ At Day 25 post‐injection (dpi), LLC‐WT mice had lost ~25% body (*Figure* 1A), ~70% fat (*Figure* 1B), and ~34% TA, ~15% GC, and ~15% QF (*Figure* 1C) muscle weight compared with untreated mice, indicating a cancer‐induced cachectic condition. These same parameters were found unchanged at 15 dpi in LLC‐WT mice (*Figure* 1A–C). Similarly, the analysis of myofiber size distribution in TA muscles showed a 30% average reduction of CSA in LLC‐WT compared with untreated mice at 25 dpi (*Figure* 1D and *Figure* *S1*A), whereas LLC‐WT CSA was unchanged at 15 dpi. Interestingly, TA, GC, and QF muscles of cachectic mice showed increased levels of the RAGE gene (Ager) already at 15 dpi (*Figure* 1E) and Ager up‐regulation increased in muscles during muscle wasting progression (*Figure* 1E). The re‐expression of RAGE at 15 and 25 dpi in TA muscles of LLC‐WT mice was confirmed by WB and IHC analysis, respectively (*Figure* 1F,G). By double IF staining RAGE was detected in atrogin‐1‐positive (atrogin‐1^+ve^) (i.e. atrophic) TA myofibers of LLC‐WT mice at 25 dpi (*Figure* 1H).

Serum levels of the RAGE ligands, S100B, and HMGB1 also were robustly increased in LLC‐bearing compared with untreated mice (*Figure* 1I), likely released from tumour cells. Indeed, conditioned medium derived from LLC cells (LLC‐CM) or LLC tumour masses (LLC‐TM) contained significant amounts of S100B and HMGB1 (*Figure* *1*J). Similar results were obtained in C26‐bearing BALB/c mice (*Figure* *S1* *B*–E).

Thus, RAGE re‐expression in muscles of LLC‐bearing mice precedes the reduction of myofiber CSA and the loss of muscle, body, and fat mass, and RAGE overexpression is concomitant with increased serum levels of S100B and HMGB1 released by tumour cells, suggesting a role for RAGE signalling in the development of cancer cachexia.

### 3.2 Absence of RAGE prevents cancer‐induced cachexia and prolongs survival of Lewis lung carcinoma tumour‐bearing mice

**Figure 2:**
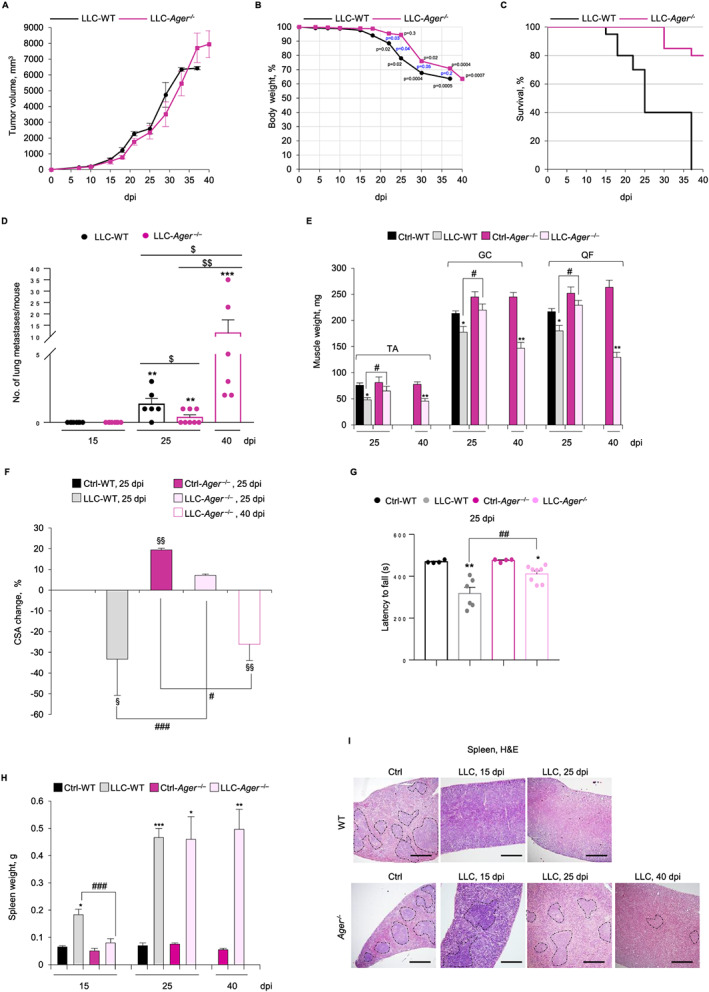
Depletion of receptor for advanced glycation end‐products (RAGE) prevents cancer‐induced cachexia, prolongs survival and maintains spleen morphology in Lewis lung carcinoma (LLC) tumour‐bearing mice. (*A*–*I*) Wild type (WT) and *Ager* ^−/−^ mice (*n* = 15 each group) injected with LLC cells were monitored for tumour growth (*A*), changes in body weight (B), and survival rates (C) until 40 dpi and sacrificed at the indicated dpi. *(D)* The number of lung metastases/mouse was determined (see also Figure *S2*C) over time in LLC‐WT and LLC*‐* *Ager* ^−/−^ mice (*n* = 8 each group). (*E*) Skeletal muscles were excised and weighed. (*F*) Reported is the average of percentage changes of tibialis anterior (TA) of the cross‐sectional area (CSA) for each mouse model compared to control WT mice (see also Figure *S3*A,B). (*G*) Plotted are the means ± standard error of the mean (SEM) of time latencies to fall in Kondziela's inverted screen test, in which each point represents an individual mouse. Spleens were weighed (*H*), and spleen morphology was analysed by haematoxylin/eosin (H&E) staining (*I*). Dashed lines indicate the boundaries of white and red pulps. Results are means ± SEM (*A*–*F*). Statistical analysis was conducted using the two‐tailed *t*‐test (*A*,*B*,*E*–*H*) or Mann–Whitney test (*D*). Significance (P) is indicated for each time‐point starting from 22 dpi (B). ^*^ *P* < 0.05, ^**^ *P* < 0.01, and *** *P*<0.001, significantly different from internal control mice (*D,* *E*,*G*,*H*). ^$^ *P* < 0.05 and ^$$^ *P* < 0.01, significantly (*D*). ^§^ *P* < 0.05 and ^§§^ *P* < 0.01, significantly different from WT (*F*). ^#^ *P* < 0.05, ## *P*<0.01, and ^###^ *P* < 0.001 significantly different. Scale bars in (*I*), 200 μm.

To determine the role of RAGE in skeletal muscle in cancer cachexia, we used the RAGE‐null (*Ager* ^*−*/−^) mice (C57BL/6 background).^23^ WT and *Ager* ^*−*/−^ mice inoculated s.c. with LLC cells showed a similar increase in tumour size (*Figure* 2A). Histological analysis of LLC masses derived from LLC‐WT or LLC‐*Ager* ^−/−^ mice showed that the absence of RAGE did not affect the extent of necrotic and fibrotic (extremely low) areas in LLC masses (*Figure* *S2*A,B) at 15 and 25 dpi. Thus, similar if not identical numbers of actively growing cancer cells were potentially able to induce cachexia in both mouse models.

Interestingly, LLC‐WT and LLC‐*Ager* ^−/−^ mice showed a different loss of body weight over time (*Figure* 2B). LLC‐WT mice showed significantly decreased body weight (~13%) at 22 dpi with maximum decrease (~36%) at 37 dpi, whereas LLC‐*Ager* ^*−*/−^ mice significantly lost body weight starting from 30 dpi (~24%), with a ~30 and ~37% weight loss at 37 and 40 dpi, respectively (*Figure* 2B). Noteworthy, LLC‐*Ager* ^*−*/−^ mice showed a surprisingly higher survival rate compared with LLC‐WT mice, with ~80% alive LLC‐*Ager* ^*−*/−^ mice vs. no surviving LLC‐WT mice at 40 dpi (*Figure* 2C). Specifically, 60% LLC‐WT mice were found dead at 25 dpi, whereas all LLC‐*Ager* ^*−*/−^ mice survived until 28 dpi (*Figure* 2C). LLC‐*Ager* ^*−*/−^ survivors at 40 dpi were sacrificed having reached ethically determined end points. Thus, absence of RAGE delays the loss of body weight and remarkably prolongs lifespan of tumour‐bearing mice, without affecting tumour growth.

Since RAGE has been implicated in the occurrence of lung metastases,^24^ which strongly reduce the survival of LLC‐bearing mice, we investigated the occurrence and number of metastases in LLC‐*Ager* ^−/−^ mice vs. LLC‐WT mice over time. We found no lung metastases at 15 dpi in LLC‐WT or LLC‐*Ager* ^−/−^ mice (*Figure* 2D and *Figure* *S2*C). At 25 dpi, the occurrence and the number of lung metastases in LLC‐*Ager* ^*−*/−^ mice were lower than in WT mice (37.5% vs. 83.3%, and 0.37 ± 0.2 vs. 1.33 ± 0.4, respectively in LLC‐*Ager* ^−/−^ vs. LLC‐WT mice) (*Figure* 2D and *Figure* *S2*C). However, at 40 dpi, 100% of survivor LLC‐*Ager* ^−/−^ mice showed a large number (11.66 ± 5.7/mouse) of lung metastases (*Figure* 2D and *Figure* *S2*C). Thus, the reduced presence of metastases might only contribute to increase the survival rate of LLC‐*Ager* ^−/−^ mice at 25 dpi.

Since cachexia is in large part caused by a decrease in skeletal muscle mass^2^, ^3^ we weighed TA, GC, and QF muscles. At 25 dpi, muscles of LLC‐WT but not LLC‐*Ager* ^*−*/−^ mice weighed significantly less than those of untreated internal controls (*Figure* 2E), suggesting that absence of RAGE confers protection against cancer‐induced muscle wasting during the first 25 dpi. However, at 40 dpi, a ~50% weight decrease was observed in muscles of survivor LLC‐*Ager* ^*−*/−^ mice (*Figure* 2E).

Analysis of myofiber size distribution in TA muscles at 25 dpi showed unchanged average CSA in LLC‐*Ager* ^−/−^ mice compared with their untreated control as opposed to LLC‐WT mice, which showed a 33.3% average reduction of CSA compared with their untreated control (*Figure* 2F). However, a significant prevalence of thin myofibers responsible for a 27.9% average reduction of CSA was detected in LLC‐*Ager* ^−/−^ mice at 40 dpi (*Figure* 2F and *Figure* *S3*A,B). Accordingly, LLC‐*Ager* ^−/−^ mice showed a significantly higher muscle strength compared with LLC‐WT mice at 25 dpi in Kondziela's inverted screen test (*Figure* 2G). Notably, the average CSA detected in TA muscles of control *Ager* ^−/−^ mice was significantly higher than in control WT mice (*Figure* 2F and *Figure* *S3*A,B), in agreement with the reported high basal number of large (>3500 μm^2^) myofibers in *Ager* ^−/−^ muscles.^16^

Serum cytokine profiling revealed increased levels of all the factors investigated with the exception of IL‐1α in LLC‐WT mice at 25 dpi compared with untreated controls (*Table* 1 and *Figure* *S4*). At this time point, LLC‐*Ager* ^−/−^ mice showed a serum protein pattern compatible with a reduced inflammatory state, as revealed by the significantly lower levels of IL‐3, IL‐6, IL‐9, IL‐12p40, IL‐12p70, and IL‐17A, IFNγ, and TNFα, and higher levels of IL‐1β compared with LLC‐WT. Interestingly, in LLC‐*Ager* ^−/−^ survivors at 40 dpi, the serum levels of IL‐3 and IL‐12p40, IFNγ, and TNFα remained even lower than those of LLC‐WT mice at 25 dpi (*Table* 1 and *Figure* *S4*), indicative of the perpetuation of the low inflammatory state in LLC‐*Ager* ^−/−^mice. The low level of the anti‐inflammatory cytokine, IL‐10, in LLC‐*Ager* ^−/−^ at 40 dpi might be a mirror and the consequence of a reduced inflammatory state.

**Table 1:** Serum cytokine multiplex analysis.

Analyte Sample	Fold change (%), LLC‐WT 25 dpi vs. Ctrl‐WT	Fold change (%), LLC‐*Ager* ^−/−^ 25 dpi vs. Ctrl‐*Ager* ^−/−^	Fold change (%), LLC‐*Ager* ^−/−^ 40 dpi vs. Ctrl‐*Ager* ^−/−^
IL‐1a	0.00	30.7	22.9
IL‐1b	249.4	↑942.2*	↑7505.4**
IL‐3	155 106.7	↓13561.8**	↓33 691.9**
IL‐6	10 072.2	↓3576.2*	↑21 109.5*
IL‐9	166 853.3	↓13561.8**	163 480
IL‐10	26 875.5	15622.5	↓1983*
IL‐12p40	44 174.9	↓200.3**	↓117.1**
IL‐12p70	416 460	↓111627.5*	462 033
IL‐17A	38 200	↓8110*	58 040
IFNγ	243 863.3	↓778.1**	↓9983.5**
TNFα	20 646.6	↓394.2*	↓3477.5*

Sera of LLC‐WT and LLC‐*Ager* ^−/−^ mice were analysed for the expression of cytokines by Biorad Bio‐Plex multiplex array compared with internal controls. Statistical analysis was conducted using the analysis of variance, Dunnet test.

IFN, interferon; IL, interleukin; TNF, tumour necrosis factor.

* *P* < 0.05 and

** *P* < 0.01, significantly different from LLC‐WT mice.

Increased spleen size (splenomegaly) and disruption of spleen structure caused by dramatic expansion of immune cells is typically observed in experimental cancer.^4^, ^25^ LLC‐WT, but not LLC‐*Ager* ^−/−^ mice, showed splenomegaly with a ~180% spleen weight increase compared with untreated controls at 15 dpi; however, a ~500% spleen weight increase was registered at 25 dpi in both LLC‐WT and LLC‐*Ager* ^−/−^ mice (*Figure* 2H). At 15 and 25 dpi, spleens of LLC‐WT mice showed disappeared boundaries of white and red pulps, with expanded haematopoietic red pulp (*Figure* 2I), a hallmark of systemic inflammation. In contrast, at 15 and 25 dpi, LLC‐*Ager* ^−/−^ mice showed a normal spleen morphology, which was partially conserved even at 40 dpi (*Figure* 2I). Collectively, these data suggested that RAGE sustains the inflammatory response in tumour conditions increasing the serum levels of cytokines involved in the development of cancer cachexia and that absence of RAGE delays the occurrence of lung metastases, delays loss of body and muscle mass, and prolongs survival of tumour‐bearing mice.

### 3.3 RAGE activity is critical for LLC‐induced muscle wasting

**Figure 3:**
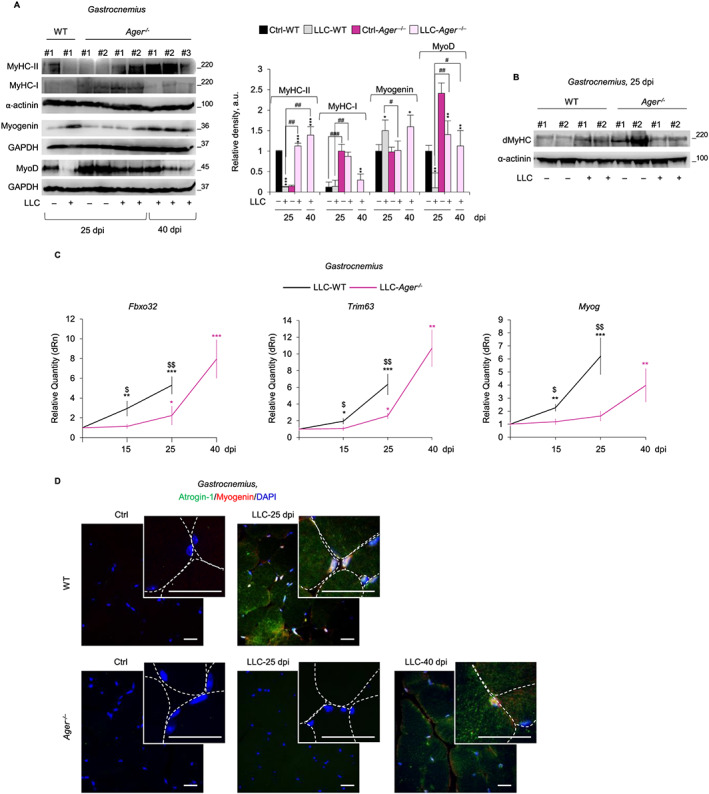
Receptor for advanced glycation end‐products (RAGE) activity is critical for Lewis lung carcinoma (LLC)‐induced muscle wasting. (*A*–*D*) *Gastrocnemius* muscles from LLC‐wild type (WT) and LLC‐*Ager* ^−/−^ mice were analysed compared with internal control mice (Ctrl) at the indicated dpi. (*A*) myoblast determination protein 1 (MyoD), myogenin and the adult fast [myosin heavy chain (MyHC)‐II] and slow (MyHC‐I) myosin heavy chain isoforms were detected by western blot (WB). Reported are the relative densities. (*B*) Expression of developmental MyHC (dMyHC) at 25 dpi was analysed by western blot (WB). α‐Actinin or GAPDH were used for loading control (*A*,*B*). (*C*) Levels of *Fbxo32*, *Trim63* and *Myog* were analysed by real‐time PCR. (*D*) Myogenin (*red*) and atrogin‐1 (*green*) were detected by immunofluorescence (IF). 4′,6‐diamidino‐2‐phenylindole (DAPI) (*blue*) was used to stain nuclei. See also Figure *S5*. Reported are high‐magnification insets with myofibers defined by dashed lines. Results are means ± standard error of the mean. Statistical analysis was conducted using the two‐tailed *t*‐test. ^*^ *P* < 0.05, ^**^ *P* < 0.01, and *** *P*<0.001, significantly different from internal control mice. ^#^ *P* < 0.05, ^##^ *P* < 0.01 and ^###^ *P* < 0.001 significantly different. ^$^ *P* < 0.05 and ^$$^ *P* < 0.01 LLC‐*Ager* ^−/−^ vs. LLC‐WT mice significantly different (*C*). Scale bars in (*D*), 25 μm.

At 25 dpi, GC and TA muscles of LLC‐WT showed strongly reduced levels of MyoD and the major sarcomeric protein, MyHC‐II, compared with untreated mice (*Figure* 3A and *Figure* *S5*A). Muscles of LLC‐*Ager* ^−/−^ showed slightly decreased MyoD and increased MyHC‐II levels at 25 and 40 dpi, indicating that absence of RAGE translates into resistance against muscle protein degradation. However, muscles of untreated *Ager* ^−/−^ mice showed remarkably higher amounts of the slow isoform MyHC‐I compared with WT muscles, and in LLC‐*Ager* ^−/−^ muscles, a robust degradation of MyHC‐I occurred at 25 and/or 40 dpi (*Figure* 3A and *Figure* *S5*A,B). Similarly, higher amounts of the developmental isoform, dMyHC, were found in muscles of untreated *Ager* ^−/−^ mice, and dMyHC appeared degraded in LLC‐*Ager* ^−/−^, but not in LLC‐WT mice, at 25 dpi (*Figure* 3B). At 15 and 25 dpi, levels of the atrogenes, *Fbxo32* (atrogin‐1) and *Trim63* (MuRF1), were significantly increased in muscles of LLC‐WT mice, as expected, whereas they were unchanged or only slightly increased in LLC‐*Ager* ^−/−^ muscles at 15 and 25 dpi, respectively, compared with their untreated controls (*Figure* 3C and *Figure* *S5*C). At 40 dpi, atrogenes in survivor LLC‐*Ager* ^−/−^ muscles reached levels comparable to those found in LLC‐WT muscles at 25 dpi. Interestingly, mRNA, and protein levels of myogenin, which has been demonstrated to induce *Fbxo32* and *Trim63* in different non cancer atrophying conditions,^8^, ^9^, ^26^ increased in LLC‐WT muscles already at 15 dpi and even more so at 25 dpi (*Figure* 3A,C and *Figure* *S5*A,C,D). Myogenin was detected in myonuclei of atrophying (atrogin^+ve^) myofibers (*Figure* 3D), as confirmed by double‐IF analysis using dystrophin as a marker of the sarcolemma (*Figure* *S5*E). We exclude that myogenin^+ve^ nuclei observed in myofibers of cachectic mice derive from muscle precursor cells because in cachexia conditions activated muscle precursor cells are unable to terminally differentiate (and to express myogenin) and fuse into myofibers.^27^, ^28^

Notably, no such changes in myogenin expression occurred in LLC‐*Ager* ^*−/−*^ muscles until 40 dpi (*Figure* 3A,C,D and *Figure* *S5*A,C). Collectively, these results suggest that tumour‐induced re‐expression of RAGE in skeletal muscles is concomitant with the activation of UPS and the up‐regulation of myogenin and that RAGE signalling is critical for muscle protein degradation.

### 3.4 RAGE signalling is required for TNFα ± IFNγ‐induced muscle atrophy

To investigate the molecular mechanism(s) underpinning the atrophying activity of muscular RAGE in cancer condition, we used two well‐characterized *in vitro* models of muscle atrophy consisting of myotube cultures exposed to TNFα alone or in combination with IFNγ. TNFα ± IFNγ is known to cause a pronounced and selective reduction of sarcomeric MyHC‐II by different and still poorly understood mechanisms.^29^, ^30^ TNFα alone induces MyHC degradation principally through the activation of UPS by enhancing atrogenes expression via p38 MAPK and/or by MyoD degradation via NF‐κB.^30^, ^31^ Myogenin is required for maximal activation of atrogenes in the presence of TNFα,^9^ whereas the combination of TNFα/IFNγ induces reduction of myotube size principally through the activation (phosphorylation) of the transcription factor, STAT3, which directly inhibits MyoD synthesis translating into reduction of MyHC‐II gene (*Myh2*) expression.^29^, ^32^ TNFα ± IFNγ also deactivates the anabolic pathway, Akt/mTOR, to reduce sarcomeric protein synthesis.^33^

**Figure 4:**
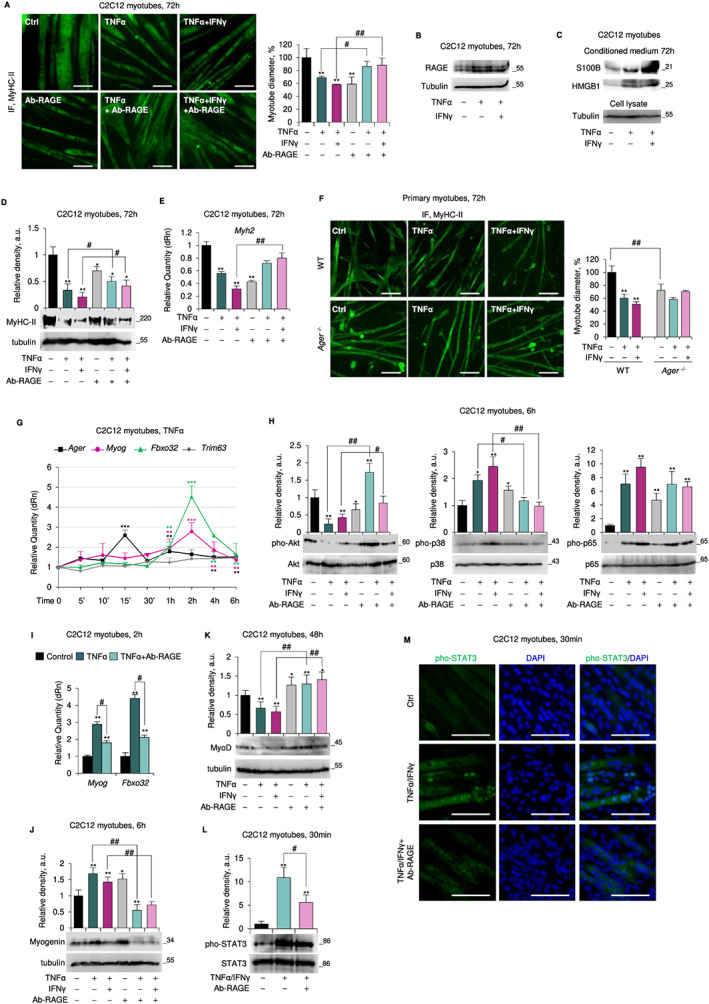
Receptor for advanced glycation end‐products (RAGE) signalling is required for tumour necrosis factor (TNF)α ± interferon (IFN)γ‐induced reduction of myotube size. (*A*–*E*, *G*–*M*) Myotubes obtained by culturing C2C12 myoblasts in differentiation medium (DM) for 4 days were added with TNFα (20 ng/ml) ± IFNγ (100 U/ml) in absence and presence of a RAGE blocking antibody (Ab‐RAGE) (10 μg/ml) for the indicated time. (*A*) Myosin heavy chain (MyHC)‐II expression was analysed by immunofluorescence (IF). Reported are the percentages of myotubes diameters compared with control. (*B*) RAGE expression was evaluated by western blot (WB). (*C*) The conditioned media of TNFα ± IFNγ‐treated myotubes were processed for detection of released S100 calcium‐binding protein B (S100B) and high mobility group box 1 (HMGB1) by WB. The tubulin is relative to cell lysates from which the media are derived. (*D*,*E*) levels of MyHC‐II protein and messenger RNA (mRNA) were evaluated by WB (*D*) and real‐time PCR (*E*). (*F*) Myotubes obtained from primary myoblasts from wild type (WT) or *Ager* ^−/−^ mice were treated with TNFα ± IFNγ to analyse MyHC‐II expression. (*G*) Levels of Ager, *Myog*, *Fbxo32*, and *Trim63* were analysed by real‐time PCR. (*H*) Total and phosphorylated protein kinase B (Akt), p38 mitogen‐activated protein kinase (MAPK), and p65 levels were analysed by western blot (WB). (*I*) Levels of *Myog* and *Fbxo32* were evaluated by real‐time PCR. (*J*,*K*) Myogenin (*J*) and myoblast determination protein 1 (MyoD) (*K*) expression was evaluated by WB. (*L*,*M*) the expression and localization of total and phosphorylated signal transducer and activator of transcription 3 (STAT3) were studied by WB (*L*) and IF (*M*). Reported are the relative densities with respect to tubulin or total form of phosphorylated protein (*D*,*H*,*J*–*L*). Results are means ± standard error of the mean (SEM) (*A*,*F*) or standard deviation (SD) (*D*,*E*,*G*–*L*). Statistical analysis was conducted using the two‐tailed *t*‐test. ^*^ *P* < 0.05, ^**^ *P* < 0.01, and ***P<0.001, significantly different from internal control. ^#^ *P* < 0.05 and ^##^ *P* < 0.01, significantly different. Scale bars (*A*,*F*,*M*), 100 μm. See also Figure *S6*,7.

Treatment of C2C12 myotubes with TNFα ± IFNγ for 72 h resulted in the reduction of myotube diameters (*Figure* 4A), increased expression of RAGE, S100B, and HMGB1, and massive S100B and HMGB1 release (*Figure* 4B,C and *Figure* *S6*A). Thus, in accordance with data obtained *in vivo* (*Figure* 1) atrophying stimuli up‐regulate RAGE in myotubes. A blockade of RAGE activity using a RAGE neutralizing antibody (Ab‐RAGE) strongly prevented TNFα ± IFNγ‐induced reduction of myotube size, as indicated by unchanged myotube diameters (*Figure* 4A) and unchanged levels of MyHC‐II protein and mRNA compared with untreated myotubes (*Figure* 4D,E).

Accordingly and consistent with *in vivo* data (*Figure* 3 and *Figure*s *S3*,*S5*), primary *Ager* ^−/−^ myotubes were resistant to TNFα ± IFNγ‐induced atrophy (*Figure* 4F and *Figure* *S6*D,E). Moreover, the pre‐treatment of C2C12 myotubes with an S100B neutralizing antibody (Ab‐S100B) together with the specific HMGB1 inhibitor, glycyrrhizin, completely protected myotubes from TNFα ± IFNγ‐induced atrophy and MyHC‐II breakdown (*Figure* *S6*B,C). Thus, S100B‐ and/or HMGB1‐activated RAGE signalling is required for induction of the atrophy programme in response to cytokine treatment.

Treatment of C2C12 myotubes with TNFα alone induced (i) a biphasic up‐regulation of RAGE and myogenin mRNA and protein (*Figure* 4G and *Figure* *S7*A); (ii) downregulation of MyoD (*Figure* *S7*A), complying with the observed reduction of *Myh2* transcription (*Figure* 4E); (iii) up‐regulation of *Myog* and *Fbxo32*, which were maximal at 2 h and preceded by Ager up‐regulation (maximal at 15 min) (*Figure* 4G); (iv) unchanged levels of *Trim63* until 6 h (*Figure* 4G); and (v) deactivation of Akt, activation p38 MAPK and NF‐κB(p65), and unchanged ERK1/2 phosphorylation levels (*Figure* 4H and *Figure* *S7*A). The activation of p38 MAPK and NF‐κB(p65) showed a main peak at 10–15 min and a further increase around 6 h concomitantly with the lowest phosphorylation extent of Akt (*Figure* *S7*A). Importantly, pharmacological inhibition of p38 MAPK by SB203580 resulted in inability of TNFα to reduce myotube diameter, degrade MyoD, and up‐regulate *Myog* and *Fbxo32* (*Figure* *S7*D‐F). In contrast, myotubes pre‐treated with the NF‐κB chemical inhibitor, BAY11‐7082, still increased *Myog* and *Fbxo32* levels in response to TNFα (*Figure* *S7*G), suggesting that myogenin‐induced UPS activation is independent of NF‐κB activity and dependent on p38 MAPK. However, MyoD degradation and the consequent reduction of myotube diameter upon treatment with TNFα was also dependent on NF‐κB activation (*Figure* *S7*D,H). Interestingly, SB203580 completely prevented the increase in TNFα‐induced Ager transcription in C2C12 myotubes, whereas BAY11–7082 was without effects (*Figure* *S7*F,G). Thus, p38 MAPK is a major inducer of RAGE expression in cytokine‐dependent muscle atrophy.

In the presence of Ab‐RAGE, TNFα was unable to (i) deactivate Akt and activate p38 MAPK (*Figure* 4H); (ii) up‐regulate myogenin (mRNA and protein) and *Fbxo32* (*Figure* 4I,J); and (iii) reduce MyoD levels (*Figure* 4K). RAGE blockade did not affect significantly the NF‐κB activation state (*Figure* 4H). Treatment of C2C12 myotubes with TNFα/IFNγ caused similar effects to those observed with TNFα alone in terms of myogenin and RAGE levels (*Figure* *S7*B,C) and activation extent of p38 MAPK, NF‐κB(p65), Akt, and ERK1/2 (*Figure* 4H and *Figure* *S7*C).

Yet, different from TNFα, TNFα/IFNγ treatment did not significantly affect atrogenes' expression (*Figure* *S7*B). After TNFα/IFNγ treatment, RAGE up‐regulation preceded STAT3 phosphorylation, with a maximum extent at 30 min, and pho‐STAT3 translocation to myonuclei leading to MyoD degradation (*Figure* 4L,M and *Figure* *S7*C). The atrophying effects of TNFα/IFNγ were unaltered by the presence of SB203580 and/or BAY11–7082 (*data not shown*), in accordance with the major role of STAT3 in the TNFα/IFNγ‐induced muscle wasting.^29^ Instead, in the presence of Ab‐RAGE, TNFα/IFNγ were unable to (i) deactivate Akt and activate p38 MAPK (*Figure* 4H); (ii) efficiently phosphorylate NF‐κB(p65) (*Figure* 4H); (iii) activate STAT3 and its translocation to myonuclei (*Figure* 4L,M); and (iv) induce MyoD degradation (*Figure* 4K). TNFα or TNFα/IFNγ did not alter *Myog* expression and pho‐p38 MAPK and MyoD levels and only slightly induced *Fboxo32* in *Ager* ^−/−^ myotubes (*Figure* *S6*D,E). Thus, RAGE signalling is required for muscle protein degradation induced by both p38 MAPK/myogenin‐activated UPS, despite the significant phosphorylation of NF‐κB, and STAT3‐dependent MyoD degradation.

To confirm these effects *in vivo*, GC muscles of WT mice were injected with TNFα/IFNγ for three consecutive days. We found (i) reduced *Myh2* levels; (ii) increased Ager, *S100b*, and *Hmgb1* levels; (iii) re‐expression of RAGE in atrogin‐1^+ve^ myofibers; and (iv) massive release of S100B and HMGB1, compared with vehicle‐injected muscles (*Figure* *S8*A‐D). CSA analysis showed a marked shift towards lower values in muscles treated with TNFα/IFNγ compared with control muscles, with a significant decrease in myofiber diameter consistent with cytokine‐induced muscle atrophy (*Figure* *S8*E, left panel). Injection of *Ager* ^−/−^ GC muscles with TNFα/IFNγ did not significantly change the myofiber distribution compared with vehicle‐injected muscles, despite a slight increase in the percentage of thin myofibers (*Figure* *S8*E, right panel). Thus, absence of RAGE prevents the atrophying effect of TNFα/IFNγ *in vivo*.

### 3.5 Dual trophic and atrophying effect of RAGE signalling in myotubes in the absence of cytokines

Treatment of C2C12 myotubes with Ab‐RAGE in the absence of cytokines resulted in dose‐dependent decrease in myotube size, MyHC‐II mRNA and protein levels, reduced activation of Akt and enhanced activation of p38 MAPK and NF‐κB(p65), and increased myogenin and *Fbxo32* and *Trim63* levels (*Figure* *S6*F–J). Reduced amounts of MyHC‐II were also found following treatment of C2C12 myotubes with Ab‐S100B or glycyrrhizin to neutralize S100B and HMGB1, respectively (*Figure* *S6*K). Thus, the physiological activity of RAGE sustained by low levels of its ligands, S100B and HMGB1, appears to be necessary for myotube trophism in normal conditions.

**Figure 5:**
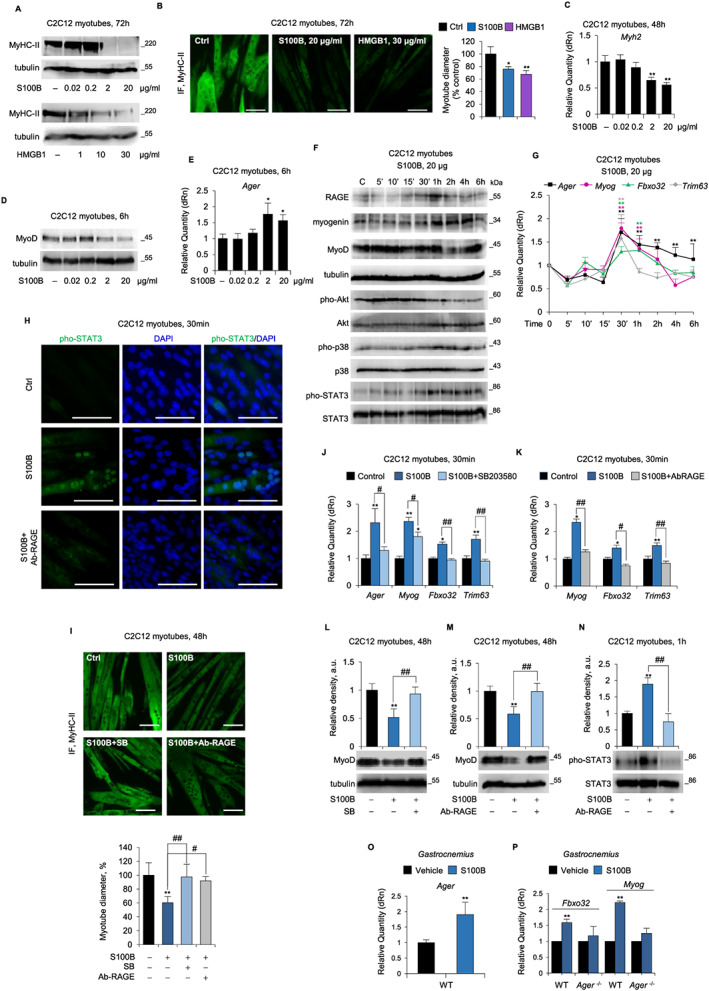
Dual trophic and atrophying effect of receptor for advanced glycation end‐products (RAGE) signalling in myotubes in the absence of cytokines. (*A*–*N*) C2C12 myotubes were cultured with S100 calcium‐binding protein B (S100B) (0–20 μg/ml) in the absence or presence of either Ab‐RAGE or SB203580, or with high mobility group box 1 (HMGB1) (0–30 μg/ml). Levels of MyHC‐II was evaluated by western blot (WB) (*A*), immunofluorescence (IF) (*B*) and real‐time PCR (*C*). Reported are the percentages of myotubes diameters relative to control (*B*). (*D*,*E*) myoblast determination protein 1 (MyoD) (*D*) and Ager (*E*) expression was analysed by western blot (WB) and real‐time PCR, respectively. (*F*) Levels of RAGE, myogenin, MyoD, and total and phosphorylated protein kinase B (Akt), p38 mitogen‐activated protein kinase (MAPK), and signal transducer and activator of transcription 3 (STAT3) were evaluated by WB. (*G*) Levels of Ager*, Myog*, *Fbxo32*, and *Trim63* were analysed by real‐time PCR. (*H*) the localization of pho‐STAT3 was evaluated by IF. (*I*) The expression of myosin heavy chain (MyHC)‐II was evaluated by WB, and the percentage of myotubes diameters respect to control was determined. (*J*,*K*) Levels of Ager (*J*) and *Myog*, *Fbxo32*, and *Trim63* (*J*,*K*) were analysed by real‐time PCR. (*L*‐*N*) Levels of MyoD protein *(L,M)* were analysed by WB, and total and phosphorylated STAT3 levels were analysed by WB (*N*). (*O*,*P*) *Gastrocnemius* muscles of WT and *Ager* ^−/−^ mice (*n* = 5 each group) were injected daily for three consecutive days with S100B (50 ng/muscle) or vehicle [phosphate‐buffered saline(PBS)] and analysed for the expression of Ager (*O*), and *Fbxo32* and *Myog* (*P*) by real‐time PCR. Results are means ± standard error of the mean (*B*,*I*) or standard deviation (*C*,*E*,*G*,*J*–*P*). Statistical analysis was conducted using the two‐tailed *t*‐test. ^*^ *P* < 0.05 and ^**^ *P* < 0.01, significantly different from internal control. ^#^ *P* < 0.05 and ^##^ *P* < 0.01, significantly different. Scale bars (*B*,*H*,*I*), 100 μm.

Administration of TNFα ± IFNγ to Ab‐RAGE‐treated myotubes caused no major changes compared to untreated controls (*Figure* 4A), which contrasts with the results obtained using *Ager* ^−/−^ myotubes (*Figure* 4F). Interestingly, administration of TNFα to C2C12 myotubes treated with a higher (50 μg/ml) dose of Ab‐RAGE did reduce their size relative to untreated controls (*Figure* *S6*L). This suggested that while neutralizing RAGE in control C2C12 myotubes, the Ab‐RAGE dose (10 μg/ml) used in the experiment in *Figure* 4A did not completely block RAGE in cytokine‐treated myotubes likely because of the cytokine‐induced overexpression of RAGE (*Figure* 4B,G) and enhanced release of the RAGE activators, S100B and HMGB1 (*Figure* 4C). Thus, the paradoxical effect of atrophying stimuli in the presence of Ab‐RAGE in C2C12 myotubes (*Figure* 4A) might be explained by an uncomplete inhibition of RAGE activity obtained with the used dose of Ab‐RAGE. Combined together, the results in *Figure*s 4A and *S6*L suggested that reduction of RAGE activity in the presence of TNFα ± IFNγ might be beneficial to myotubes, the residual RAGE activity likely exerting trophic effects. Consistently, up to a dose of 200 ng/ml, S100B exerted trophic effects on C2C12 myotubes in standard culture conditions as evidenced by the increased levels of MyHC‐II (*Figure* 5A). However, at doses ≥2 μg/ml, S100B exerted the opposite effect, strongly reducing myotube diameter and *Myh2*, MyHC‐II, and MyoD levels (*Figure* 5A–D) in concomitance with increased Ager expression (*Figure* 5E). Similar to high S100B, relatively high doses (≥1 μg/ml) of HMGB1 strongly reduced MyHC‐II levels and myotube diameters (*Figure* 5A,B), with lower doses resulting in no significant effects (*data not shown*).

While HMGB1 induced degradation of muscle proteins in cancer conditions by the activation of the autophagy system,^34^ the role of S100B in muscle wasting remained uninvestigated. We found that high S100B doses reduced pho‐Akt levels, robustly increased pho‐p38 MAPK, pho‐STAT3, and myogenin levels (*Figure* 5F), induced pho‐STAT3 nuclear translocation (*Figure* 5H), and up‐regulated *Fboxo32* and *Trim63* levels (*Figure* 5G) in C2C12 myotubes, with no significant effects on NF‐κB(p65) and ERK1/2 phosphorylation levels (*data not shown*). Notably, doses of S100B causing reduction of myotube size up‐regulated Ager expression with maximum effect at 30 min in coincidence with the maximal expression of atrogenes (*Figure* 5E,G) and preceding the maximum accumulation of RAGE protein (at 1 h) (*Figure* 5F). Thus, high S100B induces muscle protein degradation *per se* as opposed to its trophic effects at low doses.

RAGE and p38 MAPK appeared to mediate the effects of high S100B in C2C12 myotubes because Ab‐RAGE and SB203580 completely abolished high S100B's ability to induce atrophy, as assessed by measurement of myotube diameters (*Figure* 5I), *Myog* and atrogenes levels (*Figure* 5J,K), and MyoD amounts (*Figure* 5L,M). Moreover, Ab‐RAGE completely abolished high S100B's effects on STAT3 phosphorylation and nuclear translocation (*Figure* 5H,N). Interestingly, as in the case of TNFα (*Figure* *S7*F), high S100B was unable to induce Ager up‐regulation in the presence of SB203580 (*Figure* 5J), suggesting the involvement of a common p38 MAPK‐dependent mechanism. In accordance with these *in vitro* data, local injection of high S100B for two consecutive days resulted in up‐regulation of Ager, *Myog*, and *Fbxo32* levels in GC muscles of WT but not *Ager* ^−/−^ mice (*Figure* 5O,P). Collectively, these results suggested that overexpression and hyper‐stimulation of RAGE induced by high S100B might trigger muscle atrophy via a p38 MAPK/myogenin axis and STAT3‐dependent MyoD degradation, possibly amplifying the activity of cachexia‐inducing cytokines.

### 3.6 Reducing RAGE activity prevents reduction of myotube size induced by tumour‐derived factors

**Figure 6:**
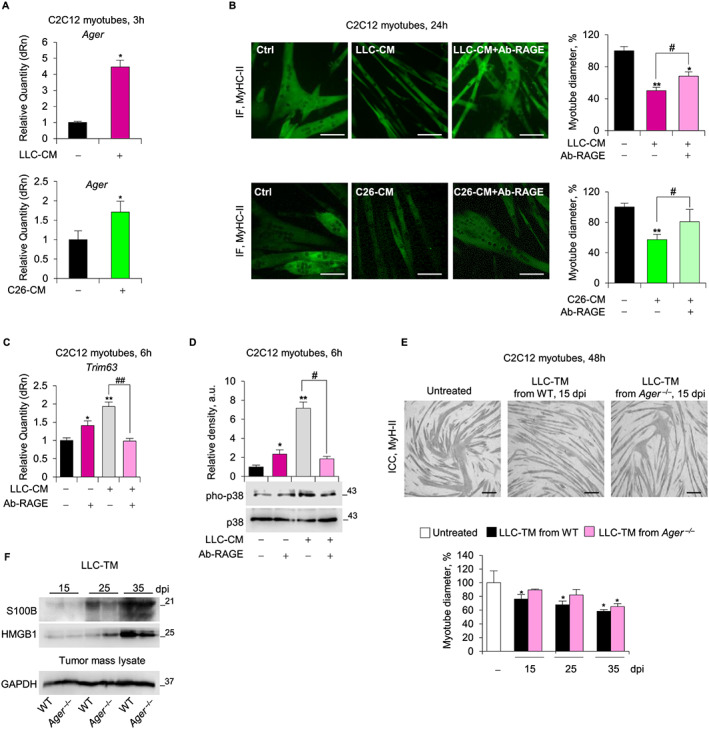
Reducing receptor for advanced glycation end‐products (RAGE) activity prevents reduction of myotube size induced by tumour‐derived factors and reduces the release of cachexia‐inducing factors from Lewis lung carcinoma (LLC) tumour. (*A*–*D*) C2C12 myotubes cultured with conditioned medium derived from LLC cells (LLC‐CM) or C26 cells (C26‐CM) in the absence or presence of Ab‐RAGE (10 μg/ml). (*A*) Ager levels were analysed by real‐time PCR. (*B*) myosin heavy chain (MyHC)‐II expression was analysed by immunofluorescence (IF), and the percent changes in myotube diameter relative to the control were determined. *Trim63* and total and phosphorylated p38 mitogen‐activated protein kinase (MAPK) were analysed by real‐time PCR (*C*) and western blot (WB) (*D*), respectively. (*E*) C2C12 myotubes were cultured with medium conditioned by LLC masses (LLC‐TM) derived from wild type (WT) or Ager^−/−^ (*n* = 5 each group) at various day post injection. Measurements of myotube diameters after MyHC‐II immunocytochemistry (ICC) analysis are shown as percentage change relative to control. (*F*) LLC‐TM derived from WT or Ager^−/−^ were analysed for S100 calcium‐binding protein B (S100B) and high mobility group box 1 (HMGB1) content by WB. GAPDH is relative to tumour lysates from which the media are derived. Results are means ± standard error of the mean (*B*,*E*) or standard deviation (*A*,*C*,*D*). Statistical analysis was conducted using the two‐tailed *t*‐test. ^*^ *P* < 0.05 and ^**^ *P* < 0.01, significantly different from internal control. ^#^ *P* < 0.05 and ^##^ *P* < 0.01, significantly different. Scale bars (*B*,*E*), 100 μm. See also Figure *S9*.

Administration of LLC‐ or C26‐CM to C2C12 myotubes recapitulated the muscle protein catabolism seen in tumour‐bearing mice through UPS activation via p38 MAPK.^35^ C2C12 myotubes treated with LLC‐ or C26‐CM showed rapid up‐regulation of Ager (*Figure* 6A) and *S100b* with unaffected levels of *Hmgb1* (*Figure* *S9*A). Treatment with Ab‐RAGE reduced tumour cell‐derived factors' ability to decrease myotube diameter (*Figure* 6B), up‐regulate *Trim63* (*Figure* 6C), and increase pho‐p38 MAPK levels (*Figure* 6D). Thus, inhibition of RAGE activity might reduce the atrophying effects of tumour‐derived factors in muscles.

*In vitro* cultured LLC cells and LLC tumour masses developed in WT or *Ager* ^−/−^ mice expressed RAGE (*Figure* *S9*B,C). MAC3^+ve^ tumour‐associated macrophages (TAMs) also expressed RAGE in WT mice (*Figure* *S9*C). Interestingly, LLC‐TM reduced C2C12 myotube diameter when derived from tumour developed in WT mice and isolated at 15 dpi but not when derived from *Ager* ^−/−^ mice until 35 dpi (*Figure* 6E). Thus, RAGE expressed in non‐tumour cells sustained the secretion of cachexia‐promoting factors from tumour masses. Interestingly, tumours developed in *Ager* ^−/−^ mice released smaller amounts of S100B, but not HMGB1, at 25 dpi compared with those derived from WT mice (*Figure* 6F), suggesting that the presence of RAGE influences the secretion of S100B from tumour masses.

## 4 Discussion

The results described herein point to a previously unravelled role of RAGE as a major player underpinning all hallmarks of cachexia under cancer conditions, that is, loss of body weight and muscle mass, systemic inflammation, and release of tumour‐derived cachexia‐inducing factors.

RAGE, which is absent in adult healthy muscle,^21^ is re‐expressed in atrophying myofibers via p38 MAPK under the action of proinflammatory cytokines and elevated serum levels of S100B and HMGB1, released from tumour cells. The re‐expression of RAGE in myofibers precedes the loss of body weight and muscle mass and strength in tumour‐bearing mice and is concomitant with the activation of muscle proteolytic UPS and the up‐regulation of myogenin, suggesting a crucial role in the onset of muscle wasting process. Indeed, in cancer conditions RAGE chronically activated by its ligands induces the activation of multiple catabolic pathways, that is, p38 MAPK and STAT3, and the deactivation of the anabolic kinase, Akt, leading at the same time to hampered MyHC synthesis and increased muscle protein degradation.

We show that myogenin is re‐expressed in the nuclei of atrophic myofibers, and we identified for the first time RAGE as a receptor able to induce myogenin expression in cachectic muscles. Muscle regeneration is highly compromised in cachexia conditions, and proliferating Pax7^+ve^ cells are unable to terminally differentiate (and to express myogenin) and fuse into myofibers because they are prevented by tumour‐derived factors influencing the muscle microenvironment. Indeed, muscles of cachectic mice typically do not show centrally‐nucleated myofibers.^27^, ^28^ Accordingly, we found higher percentages of PAX7^+ve^/ki67^+ve^ mononuclear cells in muscles of LLC‐*Ager* ^−/−^ vs LLC‐WT mice (18.3 vs. 10.2%, respectively), and absence of myogenin^+ve^ mononuclear cells in both LLC‐WT and LLC‐*Ager* ^−/−^ muscles at 25 dpi (data not shown). Thus, the myogenin^+ve^ nuclei observed in wasting conditions (*Figure*s 3D and *S5*E) are reasonably myonuclei (as identified by dystrophin localization) rather than nuclei of fused myogenin^+ve^ precursor cells, and the reduced expression of myogenin in LLC‐*Ager* ^−/−^ muscles is independent of myogenic stimuli. We speculate that the RAGE/p38 MAPK/myogenin/atrogin‐1 axis might establish a common finely regulated loop in physiological and pathological settings leading to appropriate building of muscle mass or excessive muscle protein degradation, respectively. Indeed, (i) myogenin is required for the activation of atrogin‐1, which is necessary to limit the levels of myogenic transcription factors, including myogenin itself, during both myogenesis and muscle wasting conditions (i.e. denervation, spinal muscular atrophy, and starvation)^8^, ^9^, ^26^, ^36^, ^37^; (ii) myogenin expression is regulated by RAGE signalling, and myogenin is at the same time a positive regulator of RAGE and atrogin‐1^12^, ^16^, ^38^; and (iii) chronic expression of RAGE in adult myofibers occurs in diabetes, ageing, and obesity, that is, pathological conditions characterized by muscle atrophy.^12^ Thus, RAGE, like other signalling pathways, might play distinct roles in muscle regeneration and in the balance of protein synthesis and degradation.^28^

We also demonstrate that RAGE is determinant in the muscle atrophy induced by proinflammatory cytokines and/or tumour‐derived cachexia‐inducing factors. Blocking RAGE results in inability of TNFα, TNFα/IFNγ, and tumour‐CM to activate the signalling pathways leading to muscle protein degradation.

Incidentally, our results shed light on the controversial mechanism used by TNFα to induce atrophy *in vitro*. TNFα reduces MyHC levels by inducing the degradation of MyoD, which is known to drive the transcription of structural proteins including MyHC.^37^ MyoD degradation requires two necessaries but not sufficient independent mechanisms, that is, the recruitment of a p38 MAPK/myogenin/atrogin‐1/UPS axis and the activation of NF‐κB signalling without the involvement of UPS. The fact that *Trim63*, which is able to directly degrade MyHC,^37^ is not upregulated by TNFα supports a major role of atrogin‐1‐dependent MyoD degradation in inducing myotube atrophy.

The preservation of muscle mass and a reduced occurrence of cancer metastasis have been correlated to survival in human cachectic patients.^4^, ^39^ Thus, the reduced presence of lung metastases together with the reduced muscle and body loss at 25 dpi might explain the dramatically high (80%) survival rate observed in LLC‐*Ager* ^−/−^ mice at the day of euthanasia (40 dpi) compared with LLC‐WT mice (no survivors). The higher survival rate of LLC‐*Ager* ^−/−^ mice at 40 dpi despite a dramatic loss of muscle mass and the presence of a high number of lung metastases, point to a major role of the reduction of the inflammatory state in accordance with previous work.^40^, ^41^ Indeed, survivor LLC‐*Ager* ^−/−^ mice at 40 dpi showed even lower serum levels of TNFα and IFNγ than cachectic LLC‐WT mice at 25 dpi, suggesting that these two cytokines are pivotal in determining an unfavourable prognosis. Interestingly, survivor LLC‐*Ager* ^−/−^ mice showed lower levels of the anti‐inflammatory cytokine, IL‐10. This might reflect a reduced inflammatory state and/or a reduced release of IL‐10 by RAGE‐negative TAMs in LLC masses leading to increased survival, as reported in other tumour types.^42^

In certain types of cancer, RAGE and its ligands promote spleen accumulation of myeloid‐derived suppressor cells,^43^ which have been strongly implicated in development of cancer cachexia, splenomegaly, and poor survival of cancer patients^25^ and whose activity has been correlated to serum levels of IL‐3 and IL‐17.^44^ Accordingly, splenomegaly is delayed, and the morphology of spleen is conserved even at 40 dpi in LLC‐*Ager* ^−/−^ mice, in concomitance with low serum levels of IL‐3 and IL‐17.

We demonstrate that S100B and HMGB1 are able to induce muscle atrophy *per se* at relatively high doses, behaving as cachexia‐inducing factors, and that S100B and HMGB1 are massively released from tumour masses. The combination of elevated serum S100B levels and RAGE overexpression in muscles, as seen in tumour‐bearing mice and cytokine‐induced experimental cachexia, switches the effect of the S100B‐RAGE pair from trophic to atrophying. While released HMGB1 might likewise contribute to RAGE‐induced muscle atrophy in cancer conditions, HMGB1's ability to activate TLR‐4^45^, ^46^ limits conclusions about its pro‐cachexia effects via RAGE signalling.

Surprisingly, we found that *Ager* ^−/−^ mice show a peculiar myofiber composition characterized by the persistence of developmental form of MyHC (dMyHC), a dramatic prevalence of the slow isoform, MyHC‐I, and the almost complete absence of the fast isoform, MyHC‐II, in adult healthy muscles, although the absence of RAGE does not translate into overt muscle defects.^16^ Because dMyHC and MyHC‐I precede MyHC‐II during murine muscle development,^47^ the extremely low levels of MyHC‐II together with the high levels of MyoD and dMyHC found in *Ager* ^−/−^ mice suggest an inability to achieve complete muscle maturation. The reduced content of type II myofibers, which are more vulnerable to cancer cachexia,^48^ might concur to the resistance of *Ager* ^−/−^ mice to tumour‐induced atrophy. However, an inverse shift from type I to type II myofibers occurs in the absence of RAGE in cachectic conditions, which deserves future investigation.

RAGE is a potent inducer of tumour growth and malignant conversion, migration, and invasion in many cachexia‐inducing cancer types; however, a RAGE tumour‐suppressive function has been reported in other cachexia‐inducing cancers.^11^, ^12^, ^15^ Interestingly, (RAGE^+ve^) LLC tumours developed in *Ager* ^−/−^ mice released smaller amounts of the cachexia‐promoting S100B and were less able to induce reduction of myotube size compared with LLC tumours developed in WT mice. Thus, RAGE expression and activity in tumour cells do not seem to be primarily associated with the tumour's ability to induce cachexia; cachexia might be linked to the expression of RAGE in tumour‐associated cells (such as TAMs), as well as to the presence in the serum of different RAGE ligands and likely the RAGE antagonist soluble form, sRAGE. Absence of RAGE in non‐tumour cells does not affect the extent of fibrotic and necrotic areas over time in LLC tumour masses, further supporting that the different degree of cachexia of LLC‐WT and LLC‐*Ager* ^−/−^ mice at 25 dpi is not caused by different amounts of actively growing cancer cells.

Altogether, our results suggest that targeting RAGE might represent a promising approach to counteract loss of muscle mass and strength and prolong survival in cancer patients, and measuring serum levels of the RAGE ligands, S100B and HMGB1, in non‐cachectic patients might be of prognostic value toward the future occurrence of cachexia.

## Author contributions

S.C. carried out experimental work and analysed the data; G.S. co‐designed the experiments, analysed the data and edited the manuscript; A.V. carried out *in vitro* experiments; L.S. carried out western blot analyses and in vivo experiments; R.S. analysed CM effects *in vitro*; D.C. carried out experiments with C26‐bearing BALB/c mice; G.R. performed multiplex array analysis; L.R. supervised multiplex array analysis; F.R. co‐designed the experimental work, carried out *in vivo* experiments, analysed the data and wrote the manuscript; R.D. supervised the research and edited the manuscript. All authors discussed the data and the manuscript.

## Declaration of Interest

The authors declare no competing interest.

## Supporting information

Data S1 Supporting Information
