# Supplementary material for: Targeting RAGE prevents muscle wasting and prolongs survival in cancer cachexia
Source: J Cachexia Sarcopenia Muscle. 2020 Mar 11;11(4):929–46. doi: 10.1002/jcsm.12561 (PMC7432590; doi:10.1002/jcsm.12561)
Supplement: Supplementary file 1 — Data S1 Supporting Information [file JCSM-11-929-s001.pdf]

Supplemental Material

Table S1. List of reagents and resources.

REAGENT or RESOURCE	SOURCE	IDENTIFIER
**Antibodies**		
Rabbit monoclonal anti-phospho-Akt (Ser473) (D9E)	Cell Signaling Tech.	Cat#4060
Rabbit polyclonal anti-Akt (Thr308) (D25E6)	Cell Signaling Tech.	Cat#9279
Rabbit monoclonal anti-phospho-NF-kB p65 (Ser536) (93H1)	Cell Signaling Tech.	Cat#3033
Rabbit polyclonal anti-NF-kB (p65) (C-20)	Santa Cruz Biotech.	Cat#sc-372
Rabbit monoclonal anti-phospho-p38 MAPK (Thr180/Tyr182) (D3F9) XP	Cell Signaling Tech.	Cat#4511
Rabbit monoclonal p38 MAPK (D13E1) XP	Cell Signaling Tech.	Cat#8690
Rabbit polyclonal anti-phospho-p44/42 MAPK (Erk1/2) (Thr202/Tyr204)	Cell Signaling Tech.	Cat#9101S
Rabbit polyclonal anti-MAP Kinase (ERK-1, ERK-2)	Cell Signaling Tech.	Cat#M5670
Rabbit polyclonal anti-phospho-Stat3 (Tyr705)	Cell Signaling Tech.	Cat#9131
Mouse monoclonal anti-Stat3 (124H6)	Cell Signaling Tech.	Cat#9139
Mouse monoclonal anti-MyHC-II (MF20)	eBiosciences	Cat#14-6503
Mouse monoclonal anti-MyHC-I slow (NOQ7.5.4.D)	Sigma-Aldrich	Cat#M8421
Mouse monoclonal anti-MyHC developmental (RNMY2/9D2)	Monosan	Cat#MONX10806
Rabbit polyclonal anti-Fbx32	Abcam	Cat#ab74023
Rabbit polyclonal anti-Dystrophin	Abcam	Cat#ab15277
Rat monoclonal anti-MAC3/CD107b (M3/84)	BD Biosciences	Cat#550292
Mouse monoclonal anti-MyoD (5.8A)	Santa Cruz Biotech.	Cat#sc-32758
Mouse monoclonal anti-Myogenin (F5D)	Santa Cruz Biotech.	Cat#sc-12735
Goat polyclonal anti-RAGE (N16)	Santa Cruz Biotech.	Cat#sc-8230
Mouse monoclonal anti-HMGB1 (115603)	R&D Systems	Cat#MAB1690
Mouse monoclonal anti-S100B (19/S100B)	BD Biosciences	Cat#612376
Rabbit polyclonal anti-S100B (ab41548)	Abcam	Cat#Ab41548
Mouse monoclonal anti-α-Actinin (H-2)	Santa Cruz Biotech.	Cat#sc-17829
Mouse monoclonal anti-GAPDH (6C5)	Santa Cruz Biotech.	Cat#sc-32233
Mouse monoclonal anti-α-Tubulin (DM1A)	Santa Cruz Biotech.	Cat#sc-32293
Goat anti-mouse IgG/IgM-HRP conjugated	Merck	Cat#AP130P
Goat anti-rabbit IgG-HRP conjugated	Sigma-Aldrich	Cat#A9169
Rabbit anti-goat IgG-HRP conjugated	Sigma-Aldrich	Cat#A8919
Donkey anti-Mouse IgG (H+L), Alexa Fluor 488 conjugated	Thermo Fisher Scientific	Cat#A-21202
Donkey anti-Mouse IgG (H+L), Alexa Fluor 594 conjugated	Thermo Fisher Scientific	Cat#A-21203
Donkey anti-Rabbit IgG (H+L), Alexa Fluor 488 conjugated	Thermo Fisher Scientific	Cat#A-21206

Donkey anti-Goat IgG (H+L), Alexa Fluor 594 conjugated	Thermo Fisher Scientific	Cat#A-11058
Donkey anti-Goat IgG (H+L), Alexa Fluor 488 conjugated	Thermo Fisher Scientific	Cat#A-11055
Donkey anti-Rat IgG (H+L), Alexa Fluor 594 conjugated	Thermo Fisher Scientific	Cat#A-21209
Horse anti-Goat IgG Biotinylated	Vector Laboratories	Cat#BA-9500
Horse anti-Mouse IgG Biotinylated	Vector Laboratories	Cat#BA-2000
**Chemicals, Peptides, and Recombinant Proteins**		
Recombinant bovine S100B	Our laboratory	
Recombinant HMGB1	Heikki Rauvala (Finland)	
Recombinant mouse TNF-α (*E.Coli*)	Cell Guidance Systems	Cat#GFM31
Recombinant mouse IFNγ (*E.Coli*)	Merck-Millipore	Cat#407303
SB 203580	Calbiochem	Cat#CAS 152121-47-6
BAY 11-7082	Selleckchem	Cat#S2913
Glycyrrhizic acid ammonium salt from *glycyrrhiza* root	Sigma-Aldrich	Cat#50531
Bovine Serum Albumin (BSA)	Sigma-Aldrich	Cat#A7030-100G
Blotto, non-fat dried milk	Santa Cruz Biotech.	Cat#sc-2325
DAPI dihydrochloride	Sigma-Aldrich	Cat#D 9542
VECTASTAIN® Elite® ABC HRP Kit (Peroxidase, Standard)	Vector Laboratories	Cat#PK-6100
**Critical Commercial Assays**		
ProTM Mouse Cytokine 23-plex Assay kit	Bio-Rad Laboratories	#M60-009RDPD
Mouse S100B (S100 Calcium Binding Protein B) ELISA Kit	Elabscience	Cat#E-EL-M1033
Mouse HMGB-1(High mobility group protein B1) ELISA Kit	Elabscience	Cat#E-EL-M0676
**Experimental Models: Cell Lines**		
C2C12 mouse myoblasts	ATCC, American Type Culture Collection	Cat#CRL-1772
Lewis lung carcinoma (LLC) cells	ATCC, American Type Culture Collection	Cat#CRL-1642
Colon 26 murine adenocarcinoma (C26) cells	Cell lines service (CLS)	Cat#400156
**Culture medium**		
Dulbecco's Modified Eagle's Medium (DMEM)	Gibco	Cat#41966-029
RPMI Medium 1640	Gibco	Cat#21875-034
Fetal bovine serum (FBS)	Gibco	Cat#10270-106
Horse Serum (HS)	Gibco	Cat#16050-122
Trypsin (2.5%)	Gibco	Cat#15090-046
**Experimental Models: Organisms/Strains**		
C57BL/6 mice	Charles River Laboratories	Cat#B6JSIMA06S#Z
BALB/c mice	Charles River Laboratories	Cat#C-NSIMA03S
C57BL/6 *Ager*$^{-/-}$ mice	Internal breeding	N/A
**Oligonucleotides**		
Primers *see Table S2*	This paper, Invitrogen	N/A

**Software and Algorithms**		
ImageJ software	Wayne Rasband (NIH)	https://imagej.nih.gov/ij/
xPONENT® 4.2	Luminex Corporation	
MxPro-Mx3000P v4.10	Agilent Technologies Stratagene	
Image Studio Digits v3.1.4	LI-COR	
SPOT Imaging v3.5.4	Diagnostic Instruments	
LAS (Leica Application Suite) software v4.12.0	LEICA	
IBM® SPSS® Statistics v18 software	SPSS (Chicago, IL)	
**Other**		
TRIsure™	Bioline	BIO-38033
PrimeScript™ RT reagent Kit with gDNA Eraser	Takara	RR047B
5X HOT FIREPol EvaGreen qPCR Mix Plus (ROX)	Solis BioDyne	08-24-0000
Western Bright Quantum HRP substrate	Advansta	K-12042-D20

Table S2. List of primers used in real-time PCR.

Gene	Forward primer 5'-3'	Reverse primer 5'-3'
Ager	CACTTGTGCTAAGCTGTAAGGG	CATCGACAATTCCAGTGGCTG
Fbxo32	GTCTTGGGAATGTACGACGC	GCGCTCCTTCGTACTTCCTT
Gapdh	GCCTTCCGTGTTCCTACCC	CAGTGGGCCCTCAGATGC
Hmgb1	GGCTGACAAGGCTCGTTATG	GGGCGGTACTCAGAACAGAA
Myh2	AAACAGAAGCAGGAGCGACC	CACCGGCAGCCATTTGTAAG
Myogenin	CTGGGGACCCCTGAGCATTG	ATCGCGCTCCTCCTGGTTGA
S100b	TGGCTGCGGAAGTTGAGATT	CTCACCAAGGGCTAAGCAGG
Trim63	ATTGTAGAAGCCTCCAAGGG	GGTGTTCTTCTTTACCCTCTGTG

Table S3. List of primary and secondary antibodies used in WB.

Primary Antibody	Molecular weight (kDa)	Dilution	Secondary antibody	Dilution
Goat polyclonal anti-RAGE (N16)	55	1:1000	Rabbit anti-goat IgG-HRP conjugated	1:2000
Mouse monoclonal anti-GAPDH (6C5)	37	1:1000	Goat anti-mouse IgG/IgM-HRP conjugated	1:1000
Mouse monoclonal anti-HMGB1 (115603)	25	1:1000	Goat anti-mouse IgG/IgM-HRP conjugated	1:1000
Mouse monoclonal anti-MyHC-I slow (NOQ7.5.4.D)	220	1:2000	Goat anti-mouse IgG/IgM-HRP conjugated	1:2000
Mouse monoclonal anti-MyHC-II (MF20)	220	1:10000	Goat anti-mouse IgG/IgM-HRP conjugated	1:10000

Mouse monoclonal anti-MyHC developmental (RNMY2/9D2)	220	1:500	Goat anti-mouse IgG/IgM-HRP conjugated	1:1000
Mouse monoclonal anti-MyoD (5.8A)	45	1:500	Goat anti-mouse IgG/IgM-HRP conjugated	1:1000
Mouse monoclonal anti-Myogenin (F5D)	34	1:1000	Goat anti-mouse IgG/IgM-HRP conjugated	1:1000
Mouse monoclonal anti-S100B (19/S100B)	21	1:2000	Goat anti-mouse IgG/IgM-HRP conjugated	1:1000
Mouse monoclonal anti-STAT3 (124H6)	86	1:1000	Goat anti-mouse IgG/IgM-HRP conjugated	1:1000
Mouse monoclonal anti-α-Actinin (H-2)	100	1:5000	Goat anti-mouse IgG/IgM-HRP conjugated	1:1000
Mouse monoclonal anti-α-Tubulin (DM1A)	55	1:2000	Goat anti-mouse IgG/IgM-HRP conjugated	1:2000
Rabbit monoclonal anti-p38 MAPK (D13E1) XP	43	1:2000	Goat anti-rabbit IgG-HRP conjugated	1:1000
Rabbit monoclonal anti-phospho-Akt (Ser473) (D9E)	60	1:2000	Goat anti-rabbit IgG-HRP conjugated	1:2000
Rabbit monoclonal anti-phospho-NF-kB p65 (Ser536) (93H1)	65	1:2000	Goat anti-rabbit IgG-HRP conjugated	1:2000
Rabbit monoclonal anti-phospho-p38 MAPK (Thr180/Tyr182) (D3F9) XP	43	1:2000	Goat anti-rabbit IgG-HRP conjugated	1:2000
Rabbit polyclonal anti-Akt (Thr308) (D25E6)	60	1:2000	Goat anti-rabbit IgG-HRP conjugated	1:2000
Rabbit polyclonal anti-MAP Kinase (ERK-1, ERK-2)	42/44	1:10000	Goat anti-rabbit IgG-HRP conjugated	1:10000
Rabbit polyclonal anti-NF-kB (p65) (C-20)	65	1:2000	Goat anti-rabbit IgG-HRP conjugated	1:2000
Rabbit polyclonal anti-phospho-p44/42 MAPK (Erk1/2) (Thr202/Tyr204)	42/44	1:2000	Goat anti-rabbit IgG-HRP conjugated	1:1000
Rabbit polyclonal anti-phospho-STAT3 (Tyr705)	86	1:1000	Goat anti-rabbit IgG-HRP conjugated	1:1000

Table S4. List of primary and secondary antibodies used in IF.

Primary Antibody	Dilution	Secondary antibody	Dilution

Goat polyclonal anti-RAGE (N16)	1:20	Donkey anti-Goat IgG, Alexa Fluor 594 conjugated or Alexa Fluor 488 conjugated	1:100
Mouse monoclonal anti-MyHC-II (MF20)	1:500	Donkey anti-Mouse IgG, Alexa Fluor 488 conjugated	1:100
Mouse monoclonal anti-myogenin (F5D)	1:20	Donkey anti-Mouse IgG, Alexa Fluor 594 conjugated	1:100
Rabbit polyclonal anti-atrogin-1	1:20	Donkey anti-Rabbit IgG, Alexa Fluor 488 conjugated	1:100
Rabbit polyclonal anti-dystrophin	1:20	Donkey anti-Rabbit IgG, Alexa Fluor 488 conjugated	1:100
Rabbit polyclonal anti-phospho-STAT3 (Tyr705)	1:20	Donkey anti-Rabbit IgG, Alexa Fluor 488 conjugated	1:100
Rat monoclonal anti-MAC3/CD107b (M3/84)	1:20	Donkey anti-Rat IgG, Alexa Fluor 594 conjugated	1:100

Fig. S1; related to Fig. 1.

Cachectic C26-bearing mice re-express RAGE in muscle tissue and show increased levels of RAGE ligands in serum. (A) Measurements of cross-sectional area (CSA) of *tibialis anterior* (TA) muscles of C57BL/6 mice injected with LLC cells were performed at the indicated day post injection (dpi). **(B-D)** BALB/c mice were injected s.c. with $0.5x10^6$ C26 cells or vehicle (Ctrl) (n=3 each group) and sacrificed at 15 dpi. (B) Body weight (calculated by subtracting tumor weight from total weight) and the weight of TA muscles were measured. (C) TA muscles were analyzed for RAGE expression by WB. GAPDH were used as loading control. (D) Serum levels of S100B and HMGB1 were measured by ELISA. **(E)** S100B and HMGB1 were detected in C26 cell lysates, and conditioned media derived from C26 cells (C26-CM) or C26 tumor masses (C26-TM) by WB. M, purified S100B (5ng) or HMGB1 (10ng). Shown are representative images (C,E). Results are means±SEM. Statistical analysis was conducted using the two-tailed *t*-test. *, significantly different from control (p<0.05).

Fig. S2; related to Fig. 2.

Depletion of RAGE does not affect the extent of necrotic and fibrotic areas in LLC masses delaying the occurrence of lung metastases. (A-C) WT and *Ager*$^{-/-}$ mice (n=8 each group) were injected s.c. with LLC cells and sacrificed at the indicated dpi. LLC tumor masses developed in WT and *Ager*$^{-/-}$ mice were analyzed for necrotic (asterisks and dashed lines) (A) and fibrotic (B) areas after H&E and Masson's trichrome staining, respectively. The graph in A represents the average

percentage of necrotic area. Reported are higher magnification inserts. (C) Shown are representative micrographs of H&E-stained lungs in which asterisks mark metastases. Percentages of LLC-bearing WT and *Ager*$^{-/-}$ mice displaying lung metastases are indicated. Reported are representative images (A-C). Results are means±SEM (A). Statistical analysis was conducted using the two-tailed *t*-test. **, significantly different from the respective LLC-bearing mice at 15 dpi (p<0.01), $^{###}$, significantly different (p<0.001). Scale bars (A-C), 50μm.

Fig. S3; related to Fig. 2.

Depletion of RAGE delays muscle atrophy in cancer conditions. (A,B) WT and *Ager*$^{-/-}$ (n=15 each group) were injected s.c. with LLC cells and sacrificed at the indicated dpi. Morphology of TA muscles was evaluated after H&E staining (A), and measured cross-sectional areas (CSAs) were reported for comparison in different combinations (B). Shown are representative images (A). Scale bars, 50 μm (A).

Fig. S4; related to Table 1.

Absence of RAGE reduces the inflammatory state over time in cachectic mice. Sera of WT, *Ager*$^{-/-}$, LLC-WT and LLC-*Ager*$^{-/-}$ mice were analyzed for the expression of cytokines by Biorad Bio-Plex multiplex array. Reported are the concentration (pg/ml)±SEM of the investigated cytokines.

Fig. S5; related to Fig. 3.

Absence of RAGE promotes a different type of myofiber composition and impairs the re-expression of myogenin in the myonuclei of cachectic muscles. (A-E) *Tibialis anterior* and *Gastrocnemius* muscles from WT and *Ager*$^{-/-}$ injected s.c. with LLC cells were excised and analyzed relative to the internal control at the indicated dpi. Expression of MyoD (A), myogenin (A) and adult fast (MyHC-II) and slow (MyHC-I) myosin heavy chain isoforms (A,B) was detected by WB. α-Actinin or GAPDH was used as loading control. (C) Levels of *Fbxo32*, *Trim63* and *Myog* were analyzed by real-time PCR. (D) Myogenin expression was evaluated at 15 dpi in LLC-WT mice compared with internal control. Reported is the average relative density±SEM. (E) Myogenin (*red*) and dystrophin (*green*) were detected by double IF. DAPI (*blue*) was used to stain nuclei. Shown are representative images (A,B,D,E). Results are means±SD (C). Statistical analysis was conducted using the two-tailed *t*-test. * p<0.05, ** p<0.01 and *** p<0.001 significantly different from internal control mice. $^{\$}$ p<0.05 and $^{\$\$}$ p<0.01 LLC-*Ager*$^{-/-}$ *vs* LLC-WT mice significantly different (C). Scale bars (E), 50μm.

Fig. S6; related to Fig. 4.

RAGE signaling is required for both TNFα/IFNγ-induced atrophy and trophism in myotubes. **(A)** C2C12 myotubes were added with TNFα (20ng/ml) ±IFNγ (100U/ml) for 72h and S100B and HMGB1 expression was analyzed by WB. **(B,C)** C2C12 myotubes were pre-treated for 30min with a neutralizing S100B antibody (Ab-S100B) plus the specific HMGB1 inhibitor, glycyrrhizin (GL), and added with TNFα±IFNγ for 72h. The expression of MyHC-II was evaluated by IF (B) and WB (C), and myotube diameters were determined (B). **(D,E)** Myotubes derived from WT and *Ager*$^{-/-}$ primary myoblasts were treated as in A to analyze *Myog* and *Fbxo32* levels by real-time PCR (D), and MyoD and total and phosphorylated p38 MAPK levels by WB (E). **(F-J)** C2C12 myotubes were added with different doses of Ab-RAGE (0-10μg/ml) for the indicated times. MyHC-II expression was analyzed by IF (F), real-time PCR (G) and WB (H). Reported are the percentage of myotube diameters relative to control (F). (I) Expression of total and phosphorylated Akt, p38 MAPK and p65 was detected by WB. (J) Levels of *Fbxo32* and *Trim63* were analyzed by real-time PCR. **(K)** C2C12 myotubes were treated with Ab-S100B, glycyrrhizin (GL) or DMSO for 72h and MyHC-II expression was detected by WB. (L) C2C12 myotubes were treated with TNFα in the absence or presence of Ab-RAGE (50μg/ml) for 72h. The expression of MyHC-II was evaluated by IF, and myotube diameters were determined. Representative images (A-C,E,F,H,I,K,L) and the relative densities with respect to tubulin (C,E,H) or total p38 MAPK (E) are reported. Results are means±SEM (B,F,L) or SD (C-E,G,H,J,K). Statistical analysis was conducted using the two-tailed *t*-test. * $p<0.05$, ** $p<0.01$ and *** $p<0.001$ significantly different from internal control. $^{\#\#}$ ($p<0.01$) significantly different. Scale bars (B,F,L**)**, 100μm.

Fig. S7; related to Fig. 4.

TNFα and TNFα/IFNγ induce MyHC-II breakdown in myotubes through different mechanisms. **(A-C)** C2C12 myotubes were added with TNFα (20ng/ml) ±IFNγ (100U/ml) for the indicated time. Levels of RAGE, myogenin, MyoD, and total and phosphorylated Akt, p38 MAPK, p65 and ERK1/2 (A,C), and total and phosphorylated STAT3 (C) were evaluated by WB. (B) Levels of *Ager, Myog, Fbxo32* and *Trim63* were analyzed by real-time PCR. **(D-H)** C2C12 myotubes pre-treated with SB203580 (SB) or BAY11-7082 (BAY) for 30min before treatment with TNFα were analyzed for MyHC-II by IF (D), MyoD by WB (E,H), and *Ager, Myog* and *Fbxo32* levels by real-time PCR (F,G). Reported is the percentage of myotube diameters relative to control (D). Representative images are shown (A,C-E,H). The relative densities with respect to tubulin were determined (E,H). Results are means±SEM (D) or SD (B, E-H). Statistical analysis was conducted

using the two-tailed *t*-test. * $p<0.05$ and ** $p<0.01$ significantly different from internal control. $^{\#}$ $p<0.05$ and $^{\#\#}$ $p<0.01$ significantly different. Scale bars (D), 100μm.

Fig. S8; related to Fig. 4.

RAGE signaling is required for cytokine-induced muscle wasting. (A-E) *Gastrocnemius* (GC) muscles of WT and *Ager*$^{-/-}$ mice (n=5 each group) injected daily for 3 days with TNFα (3μg/muscle)/IFNγ (5000U/muscle) or vehicle were analyzed for *Myh2* (A) and *Ager, S100b* and *Hmgb1* (B) levels by real-time PCR. (C) RAGE *(red)* was detected by IF in atrophic myofibers marked by atrogin-1 *(green)*. DAPI *(blue)* was used to stain nuclei. Shown are representative merged images. (D) GC muscles were excised and incubated in PBS for 2h at 4°C and the conditioned PBS was analyzed for the presence of S100B and HMGB1 by WB compared to untreated muscles. GAPDH is relative to muscle lysates from which the media are derived. Representative blots are shown. (E) Reported are the CSA measured after H&E staining. Indicated are the average CSA for each condition. Results are means±SD (A,B). Statistical analysis was conducted using the two-tailed *t*-test. * $p<0.05$ and ** $p<0.01$ significantly different from internal control. Scale bars (C), 50μm.

Fig. S9; related to Fig. 6.

Conditioned medium derived from LLC or C26 cells induces upregulation of *S100b* in atrophic myotubes, and RAGE is expressed in LLC cells and tumor masses. (A) C2C12 myotubes cultured in the absence or presence of LLC-CM or C26-CM were analyzed for *Hmgb1* and *S100b* levels by real-time PCR. **(B)** Lysates of LLC cells cultured in GM were analyzed for RAGE expression by WB. **(C)** LLC tumor masses developed in WT and *Ager*$^{-/-}$ mice (n=5 each group) were analyzed at 25 dpi for RAGE *(green)* and MAC3 (a marker of activated macrophages) *(red)* expression by IF. DAPI *(blue)* was used to stain nuclei. Reported are representative images (B,C). Results are means±SD (A). Statistical analysis was conducted using the two-tailed *t*-test. * $p<0.05$. Scale bars (C), 100μm.

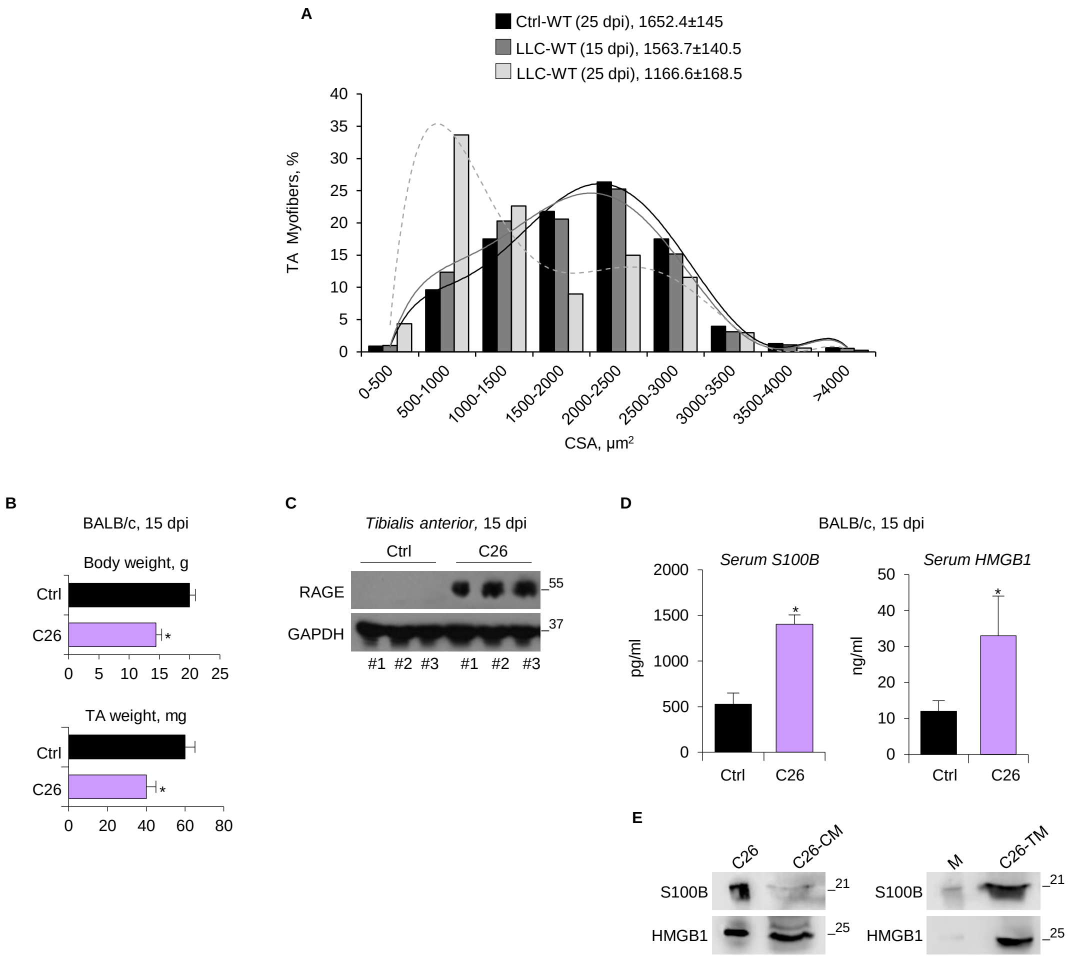
A
Ctrl-WT (25 dpi), 1652.4±145
LLC-WT (15 dpi), 1563.7±140.5
LLC-WT (25 dpi), 1166.6±168.5
TA Myofibers, %
0-500
500-1000
1000-1500
1500-2000
2000-2500
2500-3000
3000-3500
3500-4000
>4000
CSA, µm2
B
BALB/c, 15 dpi
Body weight, g
Ctrl
C26
TA weight, mg
C
Tibialis anterior, 15 dpi
Ctrl
C26
RAGE
GAPDH
#1 #2 #3 #1 #2 #3
D
BALB/c, 15 dpi
Serum S100B
Serum HMGB1
pg/ml
ng/ml
E
C26
C26-CM
M
C26-TM
S100B
HMGB1

Figure S1

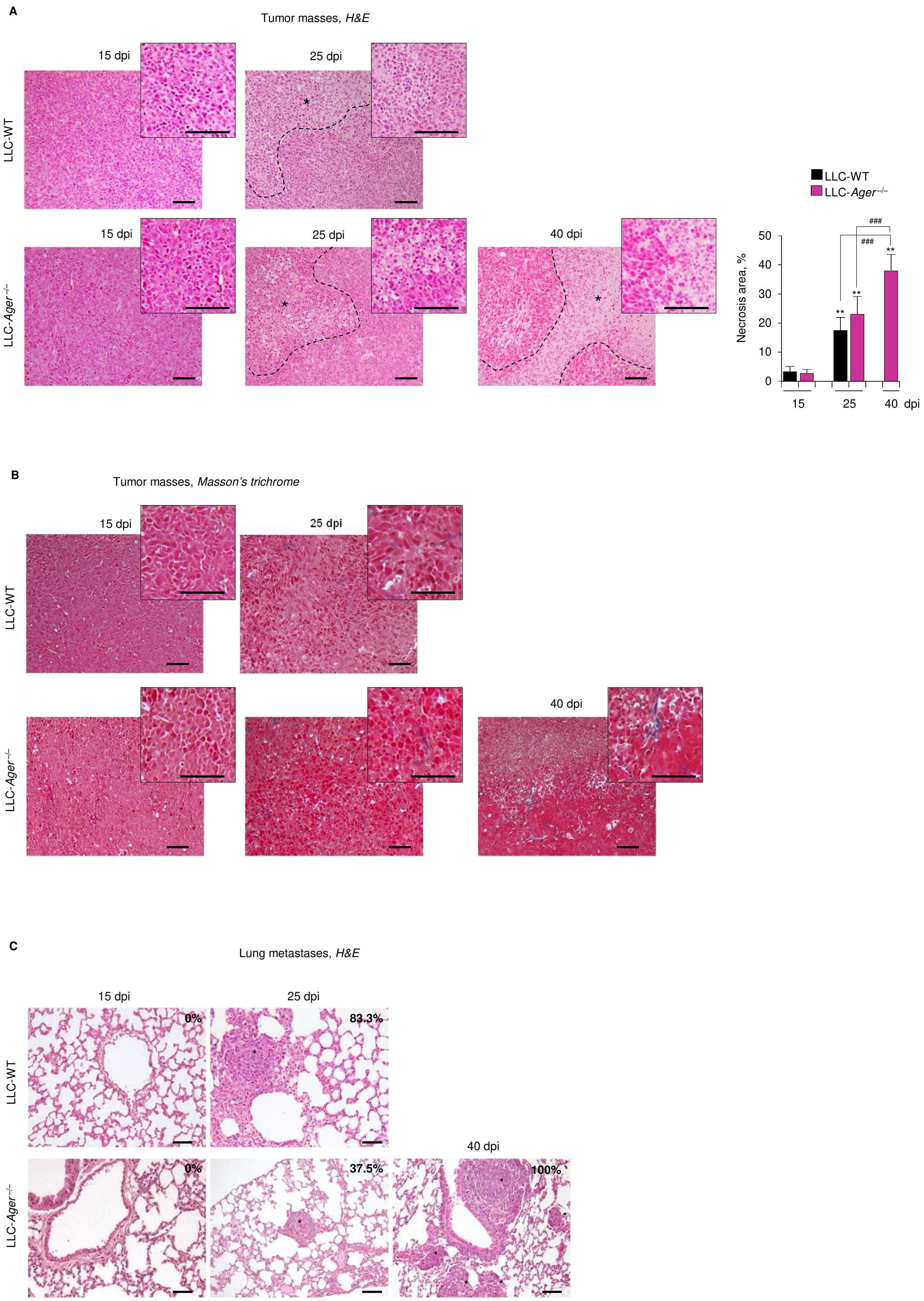
A
Tumor masses, H&E
15 dpi
25 dpi
40 dpi
LLC-WT
LLC-Ager−/−
Necrosis area, %
50
40
30
20
10
0
15
25
40 dpi
**
###
B
Tumor masses, Masson's trichrome
C
Lung metastases, H&E
0%
83.3%
37.5%
100%

Figure S2

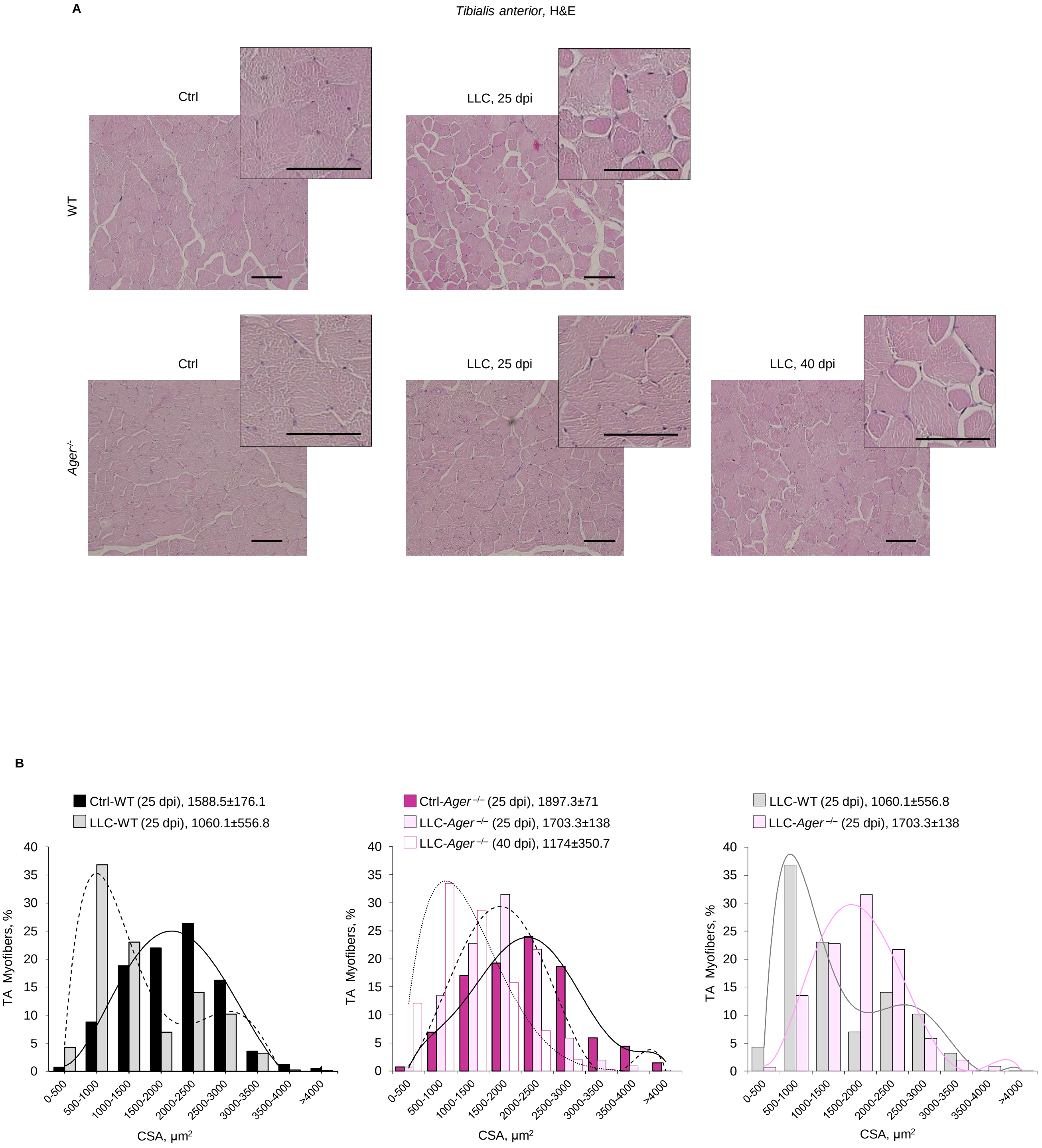
A
Tibialis anterior, H&E
Ctrl
LLC, 25 dpi
WT
Ctrl
LLC, 25 dpi
LLC, 40 dpi
Ager-/-
B
Ctrl-WT (25 dpi), 1588.5±176.1
LLC-WT (25 dpi), 1060.1±556.8
TA Myofibers, %
CSA, μm2
Ctrl-Ager−/− (25 dpi), 1897.3±71
LLC-Ager−/− (25 dpi), 1703.3±138
LLC-Ager−/− (40 dpi), 1174±350.7
TA Myofibers, %
CSA, μm2
LLC-WT (25 dpi), 1060.1±556.8
LLC-Ager−/− (25 dpi), 1703.3±138
TA Myofibers, %
CSA, μm2
0-500
500-1000
1000-1500
1500-2000
2000-2500
2500-3000
3000-3500
3500-4000
>4000

Figure S3

	Ctrl-WT	LLC-WT 25dpi	Ctrl-*Ager*$^{-/-}$	LLC-*Ager*$^{-/-}$ 25dpi	LLC-*Ager*$^{-/-}$ 40dpi
**IL-1α**	16.4±9.2	0	18.2±6.4	5.6±5.6	4.2±1.1
**IL-1β**	0.6±0.2	1.4±0.6	0.4±0.4	3.5±0.7	28±4.7
**IL-3**	0.1±0	155.1±10.5	0.2±0.1	27.1±4.11	67.4± 6.4
**IL-6**	0.4±0.1	42.8±5.4	0.4±0.1	14.3±2.8	84.4±8.3
**IL-9**	0.1±0	166.9±7.6	0.1±0	13.6±16.9	163.5±5.8
**IL-10**	0.5±0.5	122.3±55.8	2.4± 2.4	372.9±88.2	47.3± 4.4
**IL-12p40**	1.2±1.3	530.1±81.2	6.8±4.3	13.6±5.8	8.0±3.7
**IL-12p70**	0.1±0	416.5±42.5	0.1± 0	111.6±10.2	462.0±42.7
**IL-17A**	0.1±0	38.2±5.6	0.1± 0	8.1±1.1	58±18.5
**IFN-γ**	0.1±0	243.9±19	1.2±1.2	9.6±8.3	122.8±15.5
**TNF-α**	0.6±0	119.8±18	1.0±0.5	4.1±2.4	36.1±19.7

Figure S4

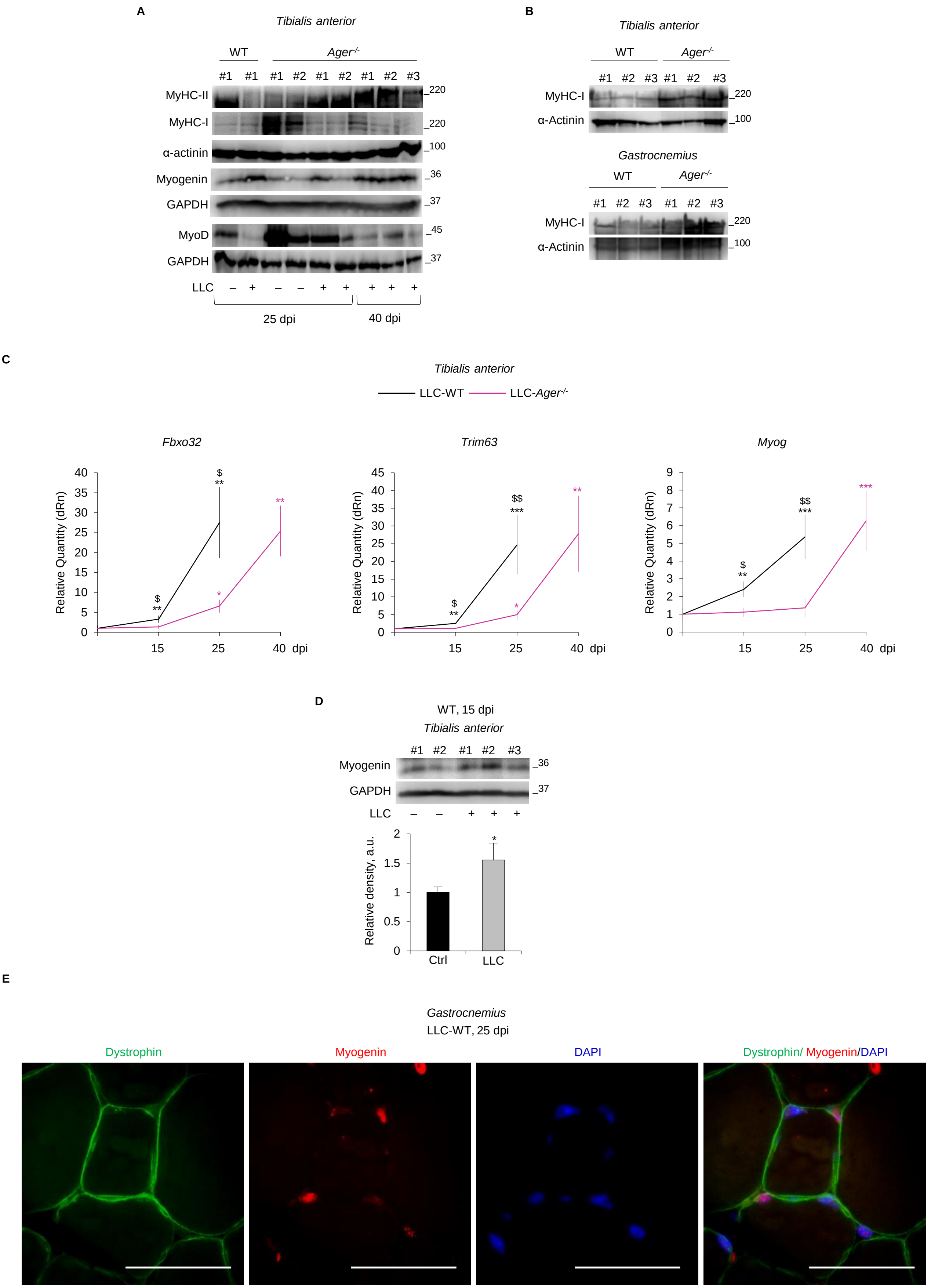

A
Tibialis anterior
WT
Ager-/-
#1 #1 #1 #2 #1 #2 #1 #2 #3
MyHC-II
MyHC-I
α-actinin
Myogenin
GAPDH
MyoD
GAPDH
220
220
100
36
37
45
37
LLC – + – – + + + + +
25 dpi
40 dpi
B
Tibialis anterior
WT
Ager-/-
#1 #2 #3 #1 #2 #3
MyHC-I
α-Actinin
220
100
Gastrocnemius
WT
Ager-/-
#1 #2 #3 #1 #2 #3
MyHC-I
α-Actinin
220
100
C
Tibialis anterior
LLC-WT
LLC-Ager-/-
Fbxo32
Trim63
Myog
Relative Quantity (dRn)
15 25 40 dpi
D
WT, 15 dpi
Tibialis anterior
#1 #2 #1 #2 #3
Myogenin
GAPDH
36
37
LLC – – + + +
Relative density, a.u.
Ctrl
LLC
E
Gastrocnemius
LLC-WT, 25 dpi
Dystrophin
Myogenin
DAPI
Dystrophin/ Myogenin/DAPI

Figure S5

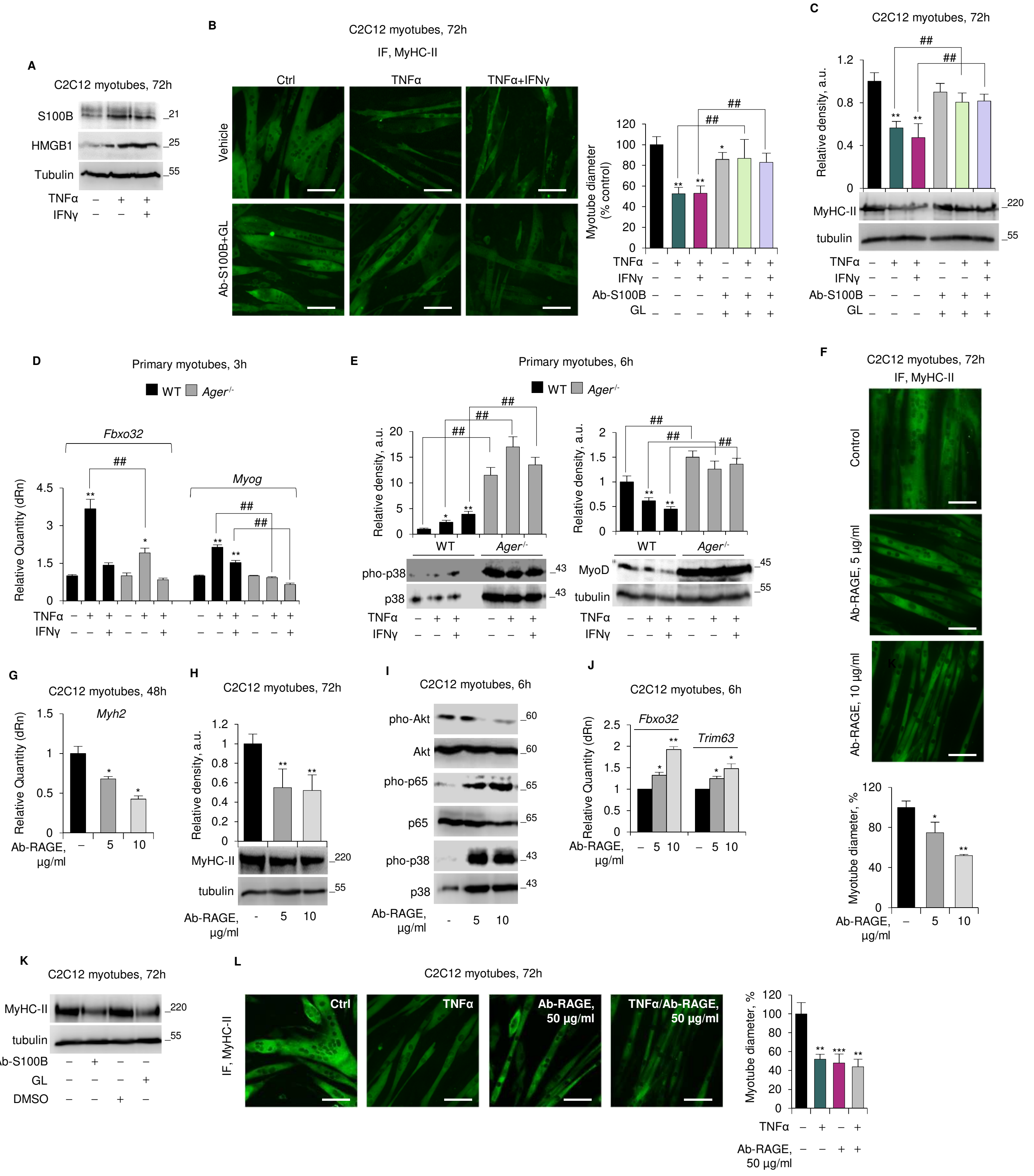
A
C2C12 myotubes, 72h
S100B
HMGB1
Tubulin
21
25
55
TNFα – + +
IFNγ – – +
B
C2C12 myotubes, 72h
IF, MyHC-II
Ctrl
TNFα
TNFα+IFNγ
Vehicle
Ab-S100B+GL
Myotube diameter (% control)
TNFα
IFNγ
Ab-S100B
GL
C
C2C12 myotubes, 72h
Relative density, a.u.
MyHC-II
tubulin
220
55
TNFα
IFNγ
Ab-S100B
GL
D
Primary myotubes, 3h
WT
Ager-/-
Fbxo32
Myog
Relative Quantity (dRn)
TNFα
IFNγ
E
Primary myotubes, 6h
WT
Ager-/-
Relative density, a.u.
pho-p38
p38
43
MyoD
tubulin
45
55
TNFα
IFNγ
F
C2C12 myotubes, 72h
IF, MyHC-II
Control
Ab-RAGE, 5 µg/ml
Ab-RAGE, 10 µg/ml
Myotube diameter, %
Ab-RAGE, µg/ml
G
C2C12 myotubes, 48h
Myh2
Relative Quantity (dRn)
Ab-RAGE, µg/ml
H
C2C12 myotubes, 72h
Relative density, a.u.
MyHC-II
tubulin
220
55
Ab-RAGE, µg/ml
I
C2C12 myotubes, 6h
pho-Akt
Akt
pho-p65
p65
pho-p38
p38
60
65
43
Ab-RAGE, µg/ml
J
C2C12 myotubes, 6h
Fbxo32
Trim63
Relative Quantity (dRn)
Ab-RAGE, µg/ml
K
C2C12 myotubes, 72h
MyHC-II
tubulin
220
55
Ab-S100B
GL
DMSO
L
C2C12 myotubes, 72h
IF, MyHC-II
Ctrl
TNFα
Ab-RAGE, 50 µg/ml
TNFα/Ab-RAGE, 50 µg/ml
Myotube diameter, %
TNFα
Ab-RAGE, 50 µg/ml

Figure S6

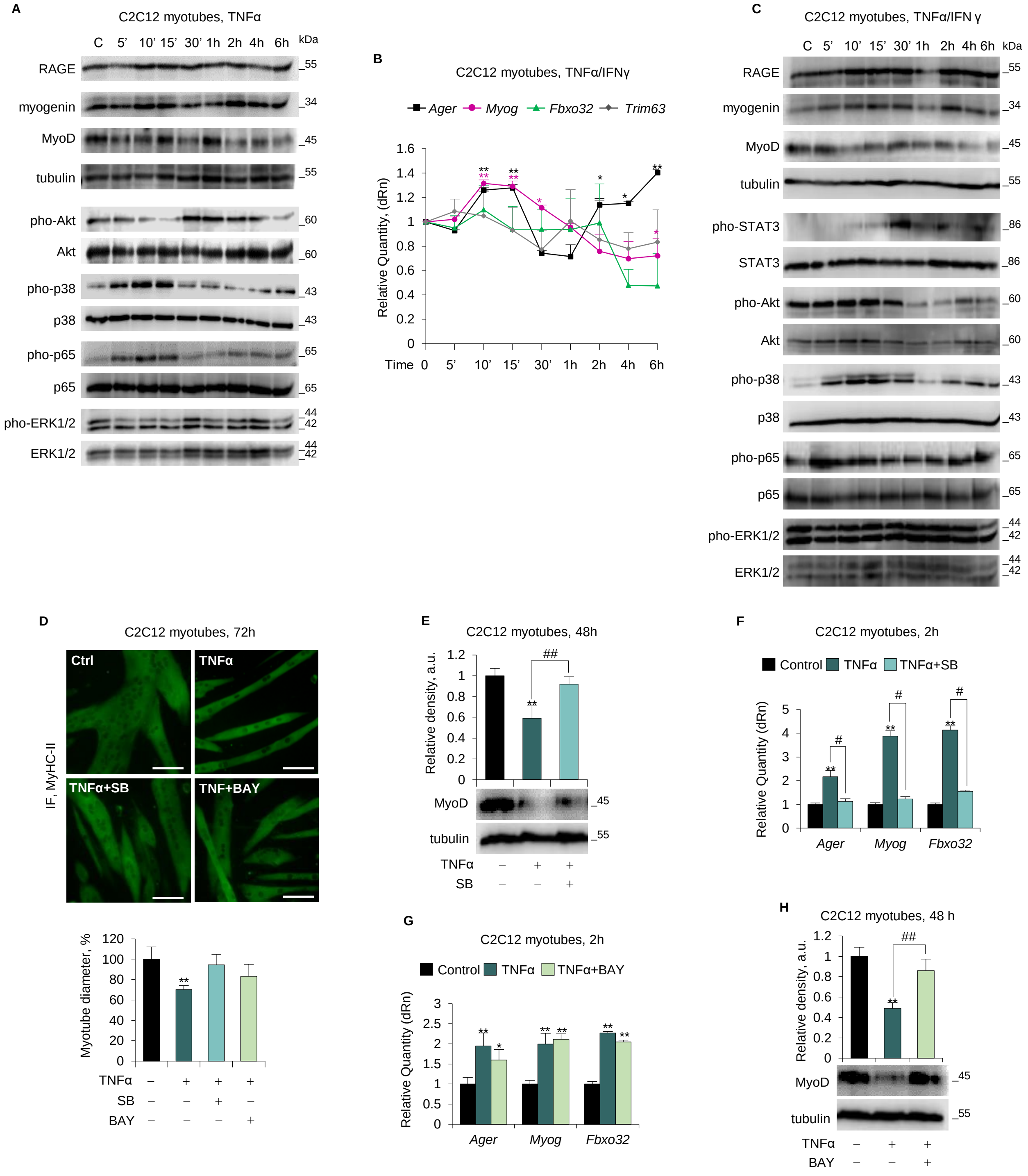

A
C2C12 myotubes, TNFα
C 5' 10' 15' 30' 1h 2h 4h 6h kDa
RAGE
myogenin
MyoD
tubulin
pho-Akt
Akt
pho-p38
p38
pho-p65
p65
pho-ERK1/2
ERK1/2
B
C2C12 myotubes, TNFα/IFNγ
Ager Myog Fbxo32 Trim63
Relative Quantity, (dRn)
Time 0 5' 10' 15' 30' 1h 2h 4h 6h
C
C2C12 myotubes, TNFα/IFN γ
C 5' 10' 15' 30' 1h 2h 4h 6h kDa
RAGE
myogenin
MyoD
tubulin
pho-STAT3
STAT3
pho-Akt
Akt
pho-p38
p38
pho-p65
p65
pho-ERK1/2
ERK1/2
D
C2C12 myotubes, 72h
Ctrl
TNFα
TNFα+SB
TNF+BAY
IF, MyHC-II
Myotube diameter, %
TNFα
SB
BAY
E
C2C12 myotubes, 48h
Relative density, a.u.
MyoD
tubulin
TNFα
SB
F
C2C12 myotubes, 2h
Control TNFα TNFα+SB
Relative Quantity (dRn)
Ager Myog Fbxo32
G
C2C12 myotubes, 2h
Control TNFα TNFα+BAY
Relative Quantity (dRn)
Ager Myog Fbxo32
H
C2C12 myotubes, 48 h
Relative density, a.u.
MyoD
tubulin
TNFα
BAY

Figure S7

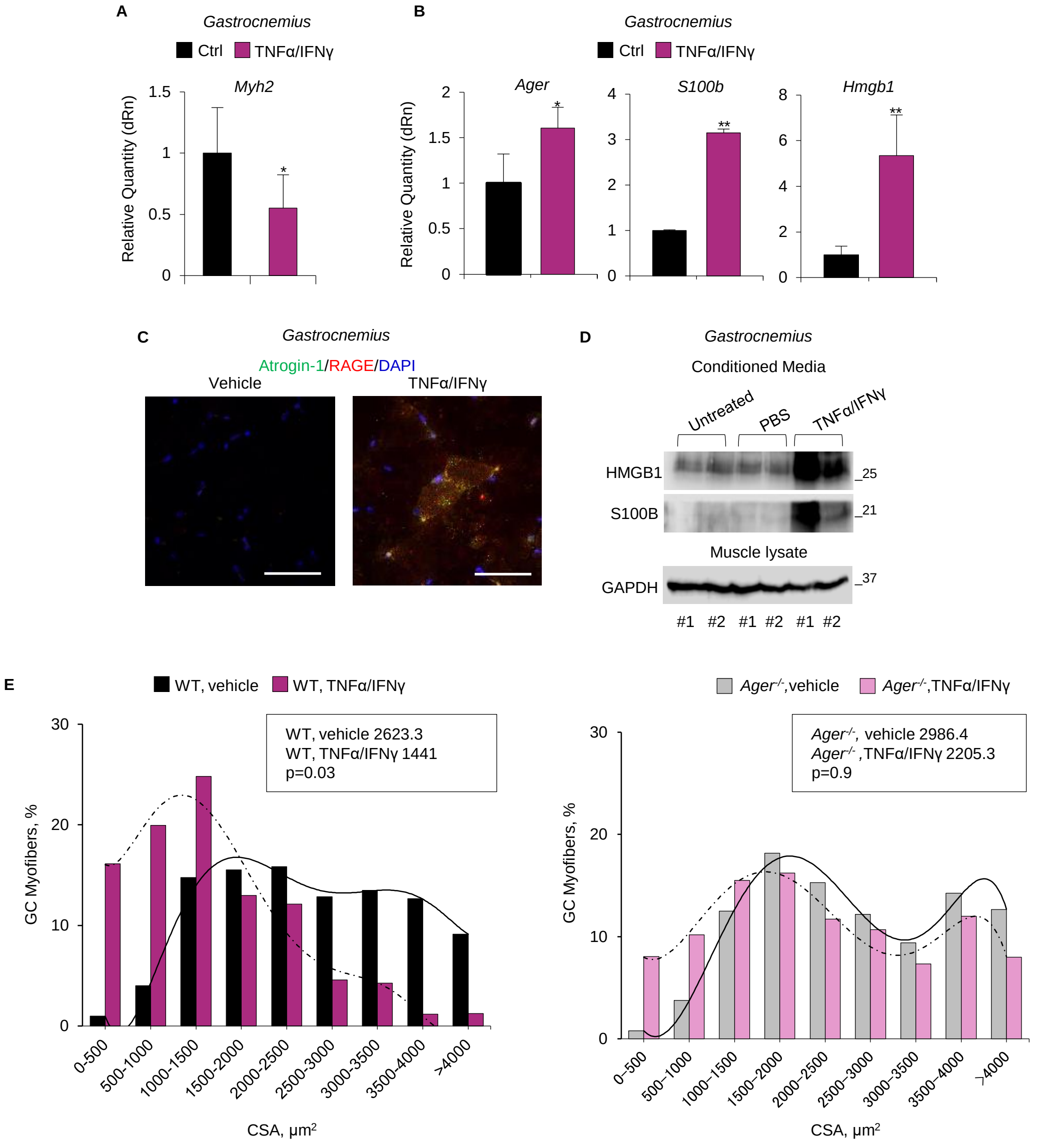
A
Gastrocnemius
Ctrl
TNFα/IFNγ
Myh2
Relative Quantity (dRn)
B
Ager
S100b
Hmgb1
C
Atrogin-1/RAGE/DAPI
Vehicle
D
Conditioned Media
Untreated
PBS
HMGB1
S100B
Muscle lysate
GAPDH
#1 #2 #1 #2 #1 #2
E
WT, vehicle
WT, TNFα/IFNγ
WT, vehicle 2623.3
WT, TNFα/IFNγ 1441
p=0.03
GC Myofibers, %
CSA, μm2
Ager-/-,vehicle
Ager-/-,TNFα/IFNγ
Ager-/-, vehicle 2986.4
Ager-/- ,TNFα/IFNγ 2205.3
p=0.9

Figure S8

A

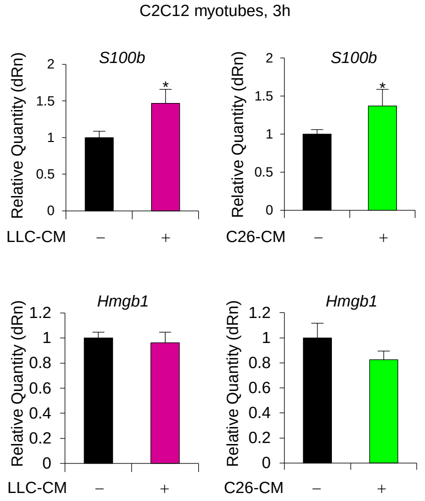

B

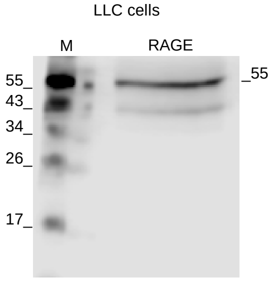

C

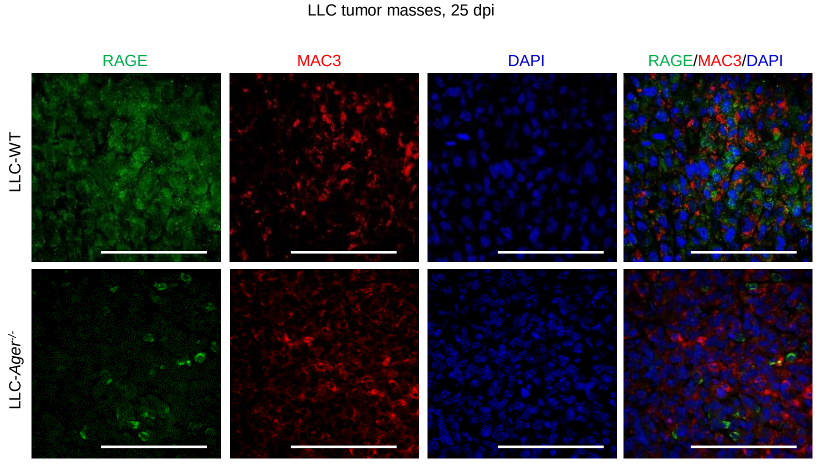

Figure S9
